# Cardiac Imaging for the Assessment of Left Atrial Mechanics Across Heart Failure Stages

**DOI:** 10.3389/fcvm.2021.750139

**Published:** 2022-01-13

**Authors:** Francesco Bandera, Anita Mollo, Matteo Frigelli, Giulia Guglielmi, Nicoletta Ventrella, Maria Concetta Pastore, Matteo Cameli, Marco Guazzi

**Affiliations:** ^1^Department of Biomedical Sciences for Health, University of Milano, Milan, Italy; ^2^Cardiology University Department, IRCCS Policlinico San Donato, Milan, Italy; ^3^Department of Cardiovascular Diseases, University of Siena, Siena, Italy; ^4^Department of Biological Sciences, University of Milano, Milan, Italy; ^5^Cardiology Division, San Paolo Hospital, Milan, Italy

**Keywords:** left atrial strain, heart failure stages, myocardial deformation, echocardiography, cardiac magnetic resonance, heart valve disease, cardiomyopathy, exercise echocardiography

## Abstract

The left atrium (LA) is emerging as a key element in the pathophysiology of several cardiac diseases due to having an active role in contrasting heart failure (HF) progression. Its morphological and functional remodeling occurs progressively according to pressure or volume overload generated by the underlying disease, and its ability of adaptation contributes to avoid pulmonary circulation congestion and to postpone HF symptoms. Moreover, early signs of LA dysfunction can anticipate and predict the clinical course of HF diseases before the symptom onset which, particularly, also applies to patients with increased risk of HF with still normal cardiac structure (stage A HF). The study of LA mechanics (chamber morphology and function) is moving from a research interest to a clinical application thanks to a great clinical, prognostic, and pathophysiological significance. This process is promoted by the technological progress of cardiac imaging which increases the availability of easy-to-use tools for clinicians and HF specialists. Two-dimensional (2D) speckle tracking echocardiography and feature tracking cardiac magnetic resonance are becoming essential for daily practice. In this context, a deep understanding of LA mechanics, its prognostic significance, and the available approaches are essential to improve clinical practice. The present review will focus on LA mechanics, discussing atrial physiology and pathophysiology of main cardiac diseases across the HF stages with specific attention to the prognostic significance. Imaging techniques for LA mechanics assessment will be discussed with an overlook on the dynamic (under stress) evaluation of the chamber.

## Introduction

The notion of the left atrium (LA) in heart failure (HF) pathophysiology has progressively moved from a by-stander chamber to a central and active element for cardiovascular balance (1, 2). The anatomical, mechanical, hemodynamical, electrical, and rheological roles of LA have been recognized and understood, especially in overt HF clinical syndrome (3, 4). The increasing availability of non-invasive approaches for LA mechanics (structural and functional properties) assessment has progressively moved the study of atrial chamber from a research interest to a clinical tool and necessity (5).

Heart failure (HF) syndrome starts with the presence of predisposing factors (stage A) and progresses with overt structural heart diseases (stage B) to a wide spectrum of clinical phenotypes (stages C and D) (6). The clinical manifestation may occur at different times of heart structural and functional changes. LA plays a major role in the physiological coupling of left ventricle (LV) filling pressures and pulmonary circulation hemodynamic (7, 8). The adaptive remodeling, aimed at contrasting volume or pressure overload and maintaining an adequate LV filling, is time and size limited. The exhaustion of compensatory mechanisms translates into overt HF or into a worsening of clinical conditions.

Cardiovascular imaging is further evolving from a morphology-based tool to a unique *in vivo* opportunity to address heart function and structure (9, 10). The understanding of LA mechanics changed with the introduction of myocardial deformation analysis and its multimodality use during stress conditions (5). The use of pharmacological or physical stressors, in order to challenge the presence of a specific ischemic, contractile, flow or diastolic “reserve” has become a standard approach with diagnostic and prognostic importance (11). The study of LA mechanics is developing in the same direction and moving toward a dynamic assessment (at rest and under stress in specific exercise) for early diagnosis and prognostic stratification of patients with HF.

The aim of the present review is to discuss the non-invasive evaluation of LA mechanics and its pathophysiological and prognostic significance across the spectrum of HF stages (including stages A and B, with the most clinically relevant structural heart diseases) with a specific focus on the available methods and the dynamic assessment. The contents will be presented according with the natural history of disease progression, starting with the conditions at risk (stage A) and proceeding with the cardiac structural diseases (stage B) and overt HF (stage C).

## LA Physiology

The LA is the inflow chamber of left-side heart and is responsible for blood accommodation from the pulmonary circulation and for diastolic LV filling (12). The peculiar attachment of pulmonary veins facilitates the blood flow during ventricular systole and isovolumetric relaxation (the so-called reservoir function) which are responsible for about 40–50% of stroke volume (SV) (13). The conduit function corresponds to the early LV diastole, or when blood flows directly from pulmonary veins to LV throughout the LA, and it accounts for the 20–30% of SV. Late diastole is characterized by active LA contraction that provides final diastolic LV filling (about 20–30% of SV). The optimal chamber function requires electromechanical synchronization, and it is strictly influenced by LV mechanical properties, transmitral gradients, and LA chamber compliance.

During exercise, the heart pumps more forcefully to generate adequate cardiac output (CO) as required by peripheral demands. At low level exercise, CO rises thanks to the SV and heart rate increase, while at heavier workload, SV maintains a plateau with chronotropic response becoming predominant (14). LA guarantees an adequate blood flow to LV during the progressive shortening of diastolic period. In normal subjects, LA volume lowers, and ejection fraction increases at an earlier stage, assuring about the 40% of flow increase with the enhancement of conduit function (15). Then, conduit and contractile phases are overlapped due to a further shortening of diastolic period, therefore, the LV filling is maintained by an additional increase of LV suction capacity during the reservoir phase.

The interplay between the active ventricular relaxation and a coherent LA response is crucial to provide adequate LV filling. Therefore, CO increase and when one or both of them fails to adapt during exertion, the unbalance causes an increase in LV filling and LA pressures, affecting the upstream pulmonary circulation (16, 17). In early pathological stages, e.g., in HF, the abnormal hemodynamic behavior can arise only during exertion, producing the typical effort-related dyspnea. In the advanced phase of the disease, the LA remodeling ends up with different degrees of enlargement, loss of function, and increase of stiffness generally associated with chronic and severe symptoms (18–20).

**Figure 1:**
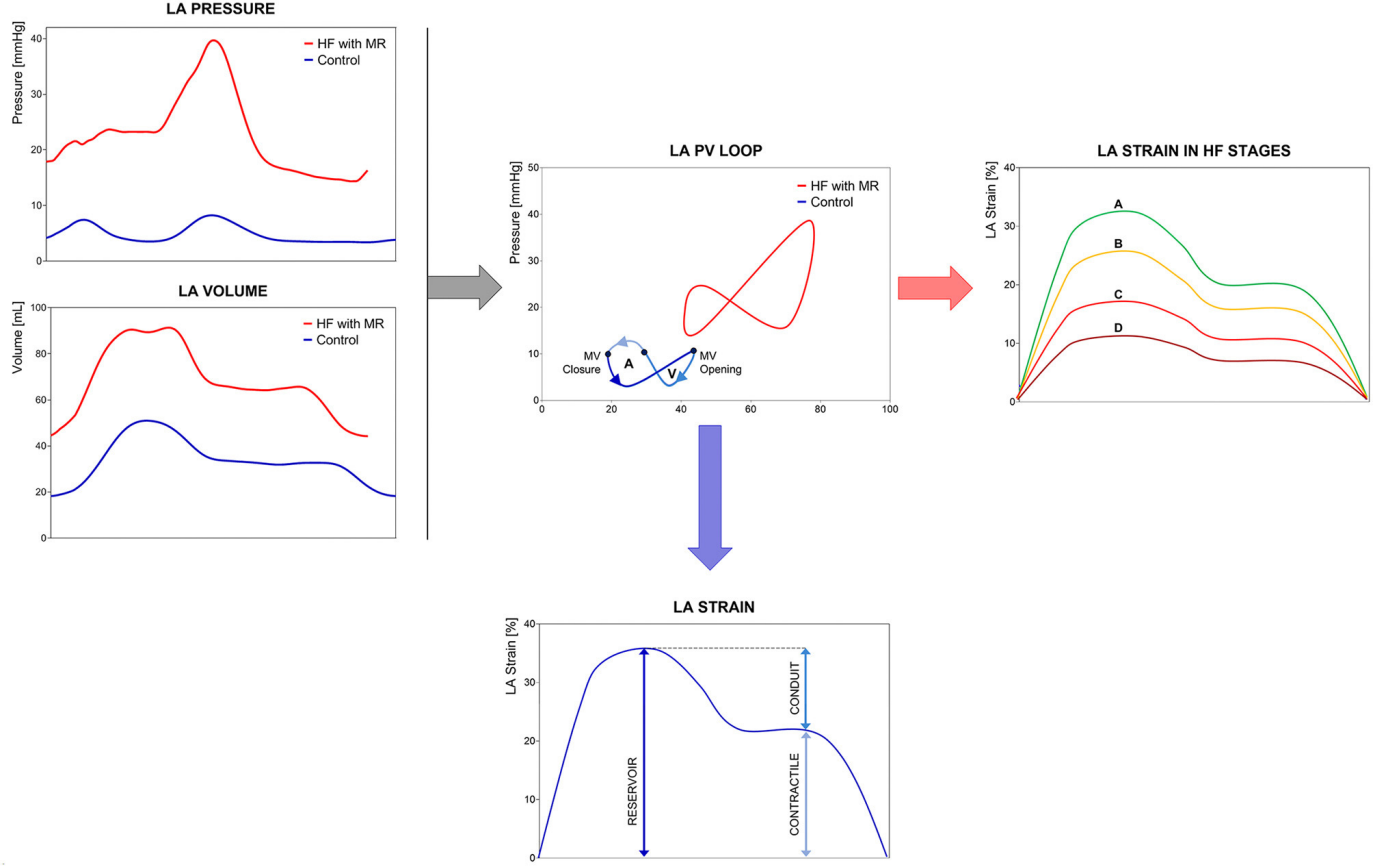
Left Atrium (LA) mechanics and physiology. Comparison of LA mechanics and physiology in a control subject vs. a heart failure with reduced ejection fraction (HFrEF) patient: **(left)** real LA pressure and volume traces in a control subject and HFrEF patient with severe mitral regurgitation; **(center)** LA Pressure-Volume loops in the same subjects; **(right)** representation of progressive LA longitudinal strain decline in the HF stages; **(bottom)** average LA longitudinal strain with three phasic components. LA pressure-volume (PV) loops show the right- and upward shifting of the loop of HF patient respect to the control subject, with loss of active atrial contraction and MR-related pressure increase. LA longitudinal strain allows the study of chamber phasic function providing physiopathological insights consistent with LA PV loop analysis. Abbreviations as in the text.

From the hemodynamic point of view, the pressure-volume (PV) loops provide a unique description of the complex physiological function, unfortunately limited by a low feasibility in clinical practice. The “eight-shaped” loop develops across the three steps of cardiac cycle, defining the active work of the chamber (left component of the loop). In HF syndrome, the PV loop shifts upward and rightward according to the chamber compliance to the pressure and volume overload (Figure 1). The use of myocardial deformation to study LA chamber represents a non-invasive technique, alternative to cardiac catheterization, and may be able to provide specific insights on chamber physiology both in control conditions and during stress conditions (physical or pharmacological).

## Cardiovascular Imaging to Evaluate LA Mechanics

**Figure 2:**
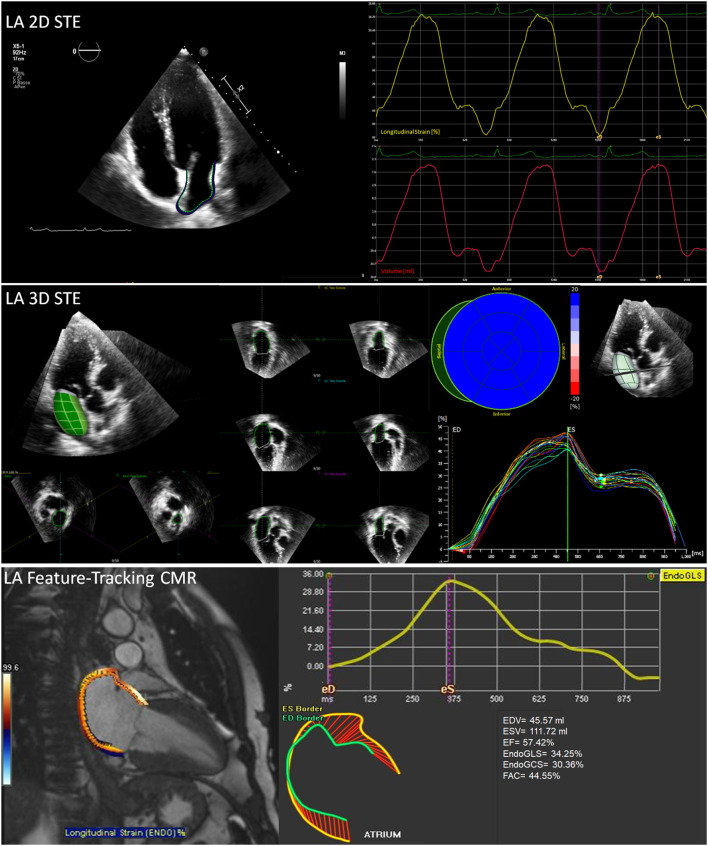
Non-invasive assessment of LA mechanics. Some elaboration and output examples of the mostly used imaging techniques are reported: **(top)** 2D speckle tracking echocardiography with atrial border contouring (top left), average longitudinal endocardial strain and volume changes **(top right)**; **(middle)** 3D speckle tracking echocardiography with 3D LA volume reconstruction **(middle left)** and multi-segment longitudinal endocardial strain; **(bottom)** feature-tracking cardiovascular magnetic resonance (CMR) with atrial border contouring **(left)** and average longitudinal endocardial strain **(bottom right)**. Abbreviations as in the text.

Transthoracic echocardiography (TTE) and cardiovascular magnetic resonance (CMR) are commonly used in clinical practice for the assessment of all cardiac chambers, including LA. Both techniques can be used at rest or under stress conditions. Two- and three-dimensional (2D and 3D) TTEs have the advantage to be widely available, feasible, and time-effective, while CMR is the reference approach for cardiac volumes quantification and tissue characterization (Figure 2).

### Parameters Describing LA Function

Myocardial deformation analysis has been applied in several clinical conditions. Table 1 provides an overview of the most relevant parameters describing LA mechanics with reference values and lower limits of normality (LLN) related to the imaging technique and with specific comments. Tables 2, 3 show the cut-off values of parameters with prognostic significance in patients at risk of HF (stage A) or with cardiac structural abnormalities (stage B) and in subjects with overt HF (stage C), respectively. Current recommendations (56) support the use of longitudinal strain due to the large amount of available literature and to the limited reliability of radial and circumferential deformation related to the low thickness of LA wall. Moreover, a global rather than regional analysis is recommended to overcome geometrical and anatomical limitations. LA function changes with the age being a dynamic instead of a static condition. Normal references are therefore presented according with age distribution when available. Figure 1 represents LA pressure, volume, and strain waveform in healthy subject and HF patient.

**Table 1:** Most relevant parameters describing left atrium (LA) mechanics with reference values and lower limits of normality (LLN).

	**Parameter**	**Definition**	**Technique**	**Normal range**	**LLN**	**Comments and accuracy**	**References**
**Echocardiography**	Left atrial expansion index (LAEI)	Relative LA volume increase during the reservoir phase (Maximal LA Volume–Minimal LA Volume)/Minimal LA Volume	2D TTE	207.1 ± 68.4 % *[mean ± SD]*	73.0	- Based on phased-related volumes analysis - Easy to calculate - Able to predict increased PAWP	(21)
	Total Emptying Fraction (TEF)	(Maximal LA Volume–Minimal LA Volume)/Maximal LA Volume	2D TTE 2D TTE 2D TTE 3D TTE	68.5 (63.2–73.2) % *[median (25*th *percentile−75*th *percentile)]* 65.8 ± 7.5 % *[mean ± SD]* 56.1 ± 12.2 % for men, 56.8 ± 12.6 % for women, p=0.21 *[mean ± SD]* 57.3 (52.4 - 61.9) % *[median (25th percentile – 75th percentile)]*	48.7 ± 1 51.1 32.2 for men, 32.1 for women 41.4 ± 1.1	- This parameter explores reservoir function based on phase-related volumes change - Time consuming - 3D TTE can provide accurate volume estimation but is limited by the presence of adequate acoustic window	(22) (21) (23) (22)
	Reservoir Strain Function	Myocardial deformation measured as difference of strain value at mitral valve opening minus ventricular end-diastole	2D STE 2D TTE 2D TTE 2D TTE 2D TTE	42.5 (36.1–48.0) % *[median (25*th *percentile−75*th *percentile)]* 45.5 ± 11.4 % *[mean ± SD]* 39.4 (33.2–46.6) % *[median (25*th *percentile−75*th *percentile)]* 35.9 ± 10.6 % *[mean ± SD]* 37.95 ± 7.96 % for men, 39.34 ± 7.99 % for women, p < 0.001 *[mean ± SD]*	26.1 ± 0 23.1 23.0 15.1 22.4 for men, 23.6 for women	- Reservoir strain is the most used parameter to evaluate the LA function - Reservoir strain function has been largely shown to be prognostic in several disease (it is related to LV systolic function) - 2D STE is entering clinical practice for its reliability but it provides information only on a single plain	(22) (21) (24) (25) (23)
**Cardiac magnetic resonance**	Total Emptying Fraction (TEF)	(Maximal LA Volume–Minimal LA Volume) / Maximal LA Volume	CMR	58.8 ± 3.7 % *[mean ± SD]*	51.5	- CMR based volumes provide very high accuracy	(26)
	Reservoir Strain Function	Myocardial deformation measured as difference of strain value at mitral valve opening minus ventricular end- diastole	MRI-FT	39.13 ± 9.27 % *[mean ± SD]*	21.0	- MRI-FT can overcome images limitations of echocardiography, but it relies on lower temporal resolution	(26)

*TTE, transthoracic echocardiography; CMR, cardiovascular magnetic resonance; 2D STE, 2D speckle-tracking echocardiography; 3D TTE, 3D transthoracic echocardiography; MRI-FT, magnetic resonance imaging–feature tracking; PAWP, Pulmonary Artery Wedge Pressure*.

**Table 2:** Summary of studies showing the prognostic value of LA mechanics in patient at risk of heart failure (HF; stage A) or with cardiac structural abnormalities (stage B) with respect to hard and soft outcomes.

**References**	**Year**	**Study population (*n*)**	**HF stage**	**NYHA class**	**Method**	**Outcome**	**LA mechanic phase**	**Predictive value**	HR or OR (^*^) (95% CI) at multivariable analysis
Paraskevaidis (27)	2009	HCM (50)	B-C	24 (48%) = I 26 (52%) = II	2D-STE	MACE 1	Reservoir	>21%	0.86 (0.77 ÷ 0.95)^*^
Roca (28)	2010	HCM (37)	B–C	12 (32%) = I 15 (41%) = II 7 (19%) = III 3 (8%) = IV	2D-STE	HF symptoms	Contractile	> −0.92 s^−1^	2.63 (1.02 ÷ 6.92)^*^
Debonnaire (29)	2013	Severe organic MR (121)	B–C	38 (32%) = I 49 (40%) = II 30 (25%) = III 4 (3%) = IV	2D-STE	Indication of MV surgery	Reservoir	≤ 24%	3.8 (1.10 ÷ 12.93)
Ancona (30)	2013	Mild to moderate rheumatic MS (101)	B	101 (100%) = I	2D-STE	AF	Reservoir	>17.4%	0.43 (0.22 ÷ 0.56)
Zito (31)	2015	Asymptomatic primary MR (67)	B	67 (100%) = I	2D-STE	Composite (All-cause mortality + AHF hospitalization + MV surgery)	Reservoir	>31.7%	0.73 (0.57 ÷ 0.93)
Yang (32)	2015	Asymptomatic primary severe MR (104)	B	70 (67%) = I 34 (33%) = Surgical class IIA indication	2D-STE	Composite (All-cause mortality + MV surgery)	Reservoir Reservoir SR	<26% <2.21 s^−1^	3.61 (1.29 ÷ 10.05)^*^ 2.86 (1.08 ÷ 7.57)^*^
Imanishi (33)	2015	Severe AS (40)	B-C	20 (50%) = I 17 (42%) = II 3 (8%) = III	2D-STE	HF symptoms	Reservoir	Per 1.0/sec increment	0.242 (0.101 ÷ 0.583)^*^
Todaro (34)	2016	Asymptomatic severe AS (82)	B	82 (100%) = I	2D-STE	Composite (All-cause mortality + AS symptoms)	Reservoir	≥ 19.8%	0.87 (0.81 ÷ 0.94)^#^
Galli (35)	2016	Severe AS (128)	B–C	50 (39%) >II	2D-STE	MACE 2	Reservoir	<21%	2.88 (1.01 ÷ 8.22)
Kamijima (36)	2017	Asymptomatic degenerative MR (91)	B	91 (100%) ≤ II	2D-STE	Exercise-induced PH	Reservoir	≤ 26.9%	NA
Modin (37)	2018	Without AF, HF, IHD (385)	A	Not reported	2D-STE	Composite (Incident IHD, HF, Cardiovascular mortality)	Reservoir	per 5% decrease	1.42 (1.01 ÷ 1.99) for women
Ring (38)	2018	Moderate to severe MR (117)	B	Not reported	2D-STE	Time to MV surgery	Reservoir Contraction	<28.5% <12.5%	3.06 (1.66 ÷ 5.61) 2.01 (1.11 ÷ 3.65)
Mohty (39)	2018	RCM (systemic AL) (77)	B–C	18 (23%) ≥III	3D-STE	All-cause mortality	Reservoir	per % increment	0.93 (0.88 ÷ 0.99)
Morris (40)	2018	Risk factor for LVDD (517)	A	Not reported	2D-STE	HF hospitalization \	Reservoir	<23%	5.7 (2.2 ÷ 14.7)^*^
Kobayashi (41)	2019	HCM (126)	B–C	96 (79%) ≤ II 25 (21%) ≥III	2D-STE	Composite (All-cause mortality + heart Tx +LV assist device implantation + clinical worsening)	Reservoir	per 1-SD decrease	2.29 (1.52 ÷ 3.48)
Vasquez (42)	2019	HCM (104)	B-C	62 (60%) = I 28 (27%) = II 14 (13%) = III	2D-STE	Composite (All-cause mortality + stroke + HF)	Reservoir Conduit	≤ 23.8% ≤ 10.2%	4.03 (1.61 ÷ 10.06) 3.64 (1.60 ÷ 8.26)
Cameli (43)	2019	Asymptomatic primary moderate MR (276)	B	Not reported	2D-STE	Composite (Cardiovascular mortality + stroke/TIA + AHF)	Reservoir	25–35% 15–25% <15%	2.5 3.2 8.6
Mateescu (44)	2019	Severe AS and preserved LV EF (248)	B–C	186 (75%) = I 59 (24%) = II 3 (1%) = IV	2D-STE	HF symptoms	Reservoir	>0.89 s^−1^	0.84 (0.73 ÷ 0.96)
Potter (45)	2020	Asymptomatic with non-ischemic HF risk factors (738)	A	Not reported	2D-STE	Incident HF	Reservoir	<24%	2.9 (1.25 ÷ 6.79)^#^
Mahfouz (46)	2020	Mild MS (75)	B	Not reported	2D-STE	Reduced exercise capacity	Reservoir	≤ 26.5%	NA
Yang (47)	2021	HCM (359)	B	Not reported	FT-CMR	Composite (Cardiovascular mortality + resuscitated CA + SCD aborted by appropriate ICD discharge + HF hospitalization)	Reservoir Conduit	≥19.5% >8.1%	0.94 (0.90 ÷ 0.99) 0.89 (0.82 ÷ 0.97)
Huntjens (48)	2021	RCM (Cardiac amyloidosis) (136)	B–C	Not reported	2D-STE	All- cause mortality	Reservoir	<13.2%	7.53 (3.87 ÷ 14.65)
Bandera (49)	2021	Transthyretin-amyloid cardiomyopathy (906)	B–C	75 (8%) = I 646 (71%) = II 179 (20%) = III 6 (1%) = IV	2D-STE	All- cause mortality	lnLA stiffness	per 1 unit increase	1.23 (1.03 ÷ 1.49)
Mandoli (50)	2021	Primary severe MR (65)	B-C	65 (100%) = II or III	2D-STE	Composite (All-cause mortality + HF)	Reservoir	≥21%	0.74 (0.58 ÷ 0.94)

* *Odds Ratio*;

# *at univariate analysis; MACE 1: Composite (Cardiac mortality + hospitalization for cardiovascular causes)*.

*Tx, transplantation; CA, cardiac arrest; SCD, sudden cardiac death; AHF, Acute heart failure; MV, Mitral Valve; MACE 2, All-cause mortality, cardiac hospitalization, and worsening HF; IHD, ischaemic heart disease; other abbreviations as in the text*.

**Table 3:** Summary of studies showing the prognostic value of LA mechanics in HF (stage C) with respect to hard and soft outcomes.

**References**	**Year**	**Study population (n)**	**HF stage**	**NYHA class**	**Method**	**Outcome**	**LA mechanic phase**	**Predictive value**	HR or OR (^*^) (95% CI) at multivariable analysis
Carluccio (51)	2018	HFrEF (405)	C	141 (35%) ≥ III	2D-STE	Composite (All-cause mortality + HF hospitalization)	Reservoir	per 1-SD decrease	1.38 (1.05 ÷ 1.84)
Lundberg (52)	2019	HF (164)	C	13 (8%) = I 27 (16%) = II 115 (70%) = III 9 (6%) = IV	2D-STE	Composite (All-cause mortality + heart Tx)	Reservoir	<21%	2.4 (1.1 ÷ 5.2)
Reddy (53)	2019	Exertional dyspnoea (363)	C	Not reported	2D-STE	HFpEF diagnosis	Reservoir	≥24.45%	0.95 (0.94 ÷ 0.97)^*^
Telles (54)	2019	Exertional dyspnoea (71)	C	2.5 ± 0.6	2D-STE	HFpEF diagnosis	Reservoir	≤ 33%	NA
Reddy (55)	2020	HFpEF (285)	C	Not reported	2D-STE	Progression to permanent AF	Reservoir	<31.5%	6.8 (3.3 ÷ 14.1)

* *Odds Ratio*.

*Tx, transplantation; other abbreviations as in the previous tables*.

### Echocardiography

The quantification of LA size has largely evolved from parasternal long axis diameter (57) to apical 4-chamber area, estimated LA volumes (58), emptying fractions (related to reservoir, conduit and contraction phases) (21, 59, 60), and 3D real-time volume (61). Notably, the usage of 2D conventional 4- and 2-chamber views (i.e., left ventricular focused) for the computation of LA size is a common source of volume underestimation, since LV and LA axes do not lie in the same plane. Hence, dedicated apical LA views should be exploited (58). The algorithm used for volume calculation represents another source of potential bias, having been shown that the area-length method provides larger volumes than Simpson disk summation (62). Nevertheless, the LA expansion index, derived by volumes estimated using Simpson disk summation, has been recently shown to predict the presence of increased pulmonary wedge pressure. In a large cohort of patients, the index has been validated with invasive right heart catheterization, showing an accuracy higher than standard echocardiographic indices (63).

The static volume has been enriched by functional assessment based on phasic changes of volume (64). However, despite the prognostic significance (65) and greater reliability of 3D vs. 2D assessment, this approach is not commonly used in clinical practice. The study of myocardial deformation, first with tissue Doppler imaging (5, 66), then with 2D speckle-tracking echocardiography (2DSTE) (23–25), has become the most widely used approach, also showing high intra and inter-individual reproducibility (67). Extensive recommendations for myocardial deformation imaging have been endorsed by the European Association of Cardiovascular Imaging (EACVI)/American Society of Echocardiography (ASE)/Industry Task Force (56) covering specific indications to standardize LA assessment. Briefly, using a non-foreshortened apical 4-chamber view with temporal resolution of at least >50 Hz, LA endocardial contour should be manually drawn (<3 mm of thickness). End-diastole, corresponding to R wave at ECG trace, is commonly used as reference since it has shown to provide a slightly higher feasibility and a lower wasting time compared to methods using atrial contraction as reference (68).

A recent meta-analysis (69) systematically reviewed 10 studies that computed pre-interventional 2DSTE LA strain capacity to predict AF recurrence in patients that underwent catheter ablation. A subgroup analysis was performed comparing studies that exploited GE EchoPac^®^ and those that used TomTec^®^ (vendor-independent software) showing that the mean strain values differed significantly between the two subgroups, both in patients with and without AF recurrence and those without. Moreover, the cut-off value predicting AF recurrences and assessed through GE Echopac (18.1%) was substantially different from the one calculated for all the studies (21.9%). A similar difference has been reported in the EACVI Normal Reference Ranges for Echocardiography (NORRE) study where the 3D LA volume and the LA strain rate significantly differed according to the different kind of vendor used. Therefore, intervendor variability should be considered in clinical setting and in the design of single and multicenter trials. Of note, all the reviewed studies were performed prior to the release of the EACVI/ASE/Industry Task Force consensus document.

Normality ranges for 2DSTE-derived LA strain have been established on a cohort of 371 healthy subjects (22) enrolled in the EACVI NORRE study and evaluated using a vendor-independent software (2D Cardiac Performance Analysis, TomTec Imaging System^®^, Munich, Germany). Multivariable analysis showed that only age is independently associated with all the LA strain components, while no differences in gender were reported. In addition, LA reservoir and conduit strain progressively decrease with age, while contractile function slightly increases (Table 1 and Supplementary Table 1).

Remarkably, 2DSTE analysis is intrinsically limited by the ability of exploring only a static bi-dimensional plan where a very mobile LA endocardium moves throughout at every beat. 3D echocardiography (3DE) is therefore emerging as an alternative approach to capture the overall LA volume and the complex motion along the three dimensions. Starting from a single LA dataset, 3DE allows the assessment of LA volumes at multiple time points during the cardiac cycle. Data from several studies (70–72) demonstrated that 3DE-derived LA volumes are more accurate and reproducible than those calculated by 2D echocardiography if compared to CMR. Only a few published studies have reported reference values of 3DE-derived LA volume. Reference values have been proposed based on a LA-dedicated software used on a cohort of 276 healthy volunteers (61) and were reported to be significantly larger (normality range 18–43 ml/m^2^) than those obtained through 2D Simpson's method, in agreement with previous reports (73). Despite the need of excellent image quality and the dependence on lower temporal resolution (frame rate > 20 volumes per second), 3DE outperforms 2D echo in the assessment of LA volume and, when available, is recommended in routine clinical practice (70, 72).

Left atrium (LA) mechanics can be studied with 3D speckle-tracking (3DSTE) algorithms implemented in commercial software. These tools compute LA strain along three spatial dimensions (longitudinal, circumferential, and radial), thus leading to deformation values that are truly able to assess the complex 3D motion of the chamber. Different studies (74–76) reported 3DSTE as a faster and more reproducible method than 2DSTE for the measurement of LA strain. The ability of 3DSTE to identify LA functional impairments has been shown in patients affected by type 1 diabetes mellitus (77), hypertrophic cardiomyopathy (78), hypertension (79), amyloidosis (39), and inappropriate sinus tachycardia (80). Few studies addressed the normal values of 3DSTE-derived LA longitudinal strain on healthy people reporting lower values when compared with 2DSTE (75, 76).

A specific strength of echocardiography is represented by its application during physical exercise. If the patient is capable of exercise, stress echocardiography can be performed with a treadmill or a cycle ergometer (upright or supine). With treadmill, the Bruce protocol is followed and images are acquired at rest, immediately after peak exercise, and at recovery (81). Using tiltable ergometer, images are continuously acquired at baseline, at each 25 W step, at peak stress, and during recovery (82). To be successful, bicycle stress tests need the cooperation of the patient and the perfect coordination of the clinicians. In most of the cases, test interpretation is then performed through the comparison of resting and peak images (83).

In order to specifically assess the LA function during stress test, images should be acquired with dedicated 4- and 2-chamber views at rest and under exercise, optimizing the sector width and depth. At baseline, the frame rate should be at least 60–70 per second, while 80–90 per second during exercise, compensating for the heart rate increase. Septal e', lateral e', and E wave of mitral inflow should be measured to allow the atrial stiffness estimation according to the formula E/e'/LA reservoir function. Evaluating the atrial function at rest and during exercise with this method is considered reliable and efficient to detect changes in atrial stiffness (84). Specific attention should be paid to E and A waves fusion occurring at HR >100–110 beats per minute. The current European consensus for the diagnostic workup of heart failure with preserved ejection fraction (HFpEF) indicates the use of exercise echocardiography when the diagnostic score is not conclusive. The recommendation consists in measuring E/e' at earlier stages of exercise (when E and A are still separated) or during recovery when E and A are no longer fused (85).

### Cardiovascular Magnetic Resonance

Cardiovascular magnetic resonance (CMR) is characterized by a high reproducibility and good non-isotropic spatial resolution (in clinical practice slices have 8 mm of thickness with in-plane resolution of 1.5–2.5 mm). Steady State Free Precession (SSFP) provides a greater endocardial signal compared to echocardiography due to the excellent blood-endocardium contrast. Nevertheless, the echocardiographic in-plane resolution can be greater according with the used ultrasound frequency (86). Acquiring unseparated slices encompassing the entire LA during the whole cardiac cycle allows the measurement of LA SV, ejection fraction, and volumes during the whole cardiac cycle, providing phase-related emptying fractions (as described for echocardiography). This approach has the unique strength of measuring real volumes with the highest accuracy, also in very remodeled chambers. However, since Simpson's method is time consuming, the biplane area-length method, which is based on the manual tracing of the LA walls in cine-sequences of 4- and 2-chamber views, it is more frequently used despite a possible underestimation related to non-LA dedicated slices (87).

A unique feature of CMR is the identification of scar with gadolinium and its use is a routine practice in LV evaluation, while the thinness of LA wall does not allow a common and wide application. Nevertheless, the Delayed-Enhancement MRI Determinant of Successful Radiofrequency Catheter Ablation of Atrial Fibrillation (DECAAF) study, a multicenter study conducted at 15 different clinical centers (88), showed the feasibility of LA assessment, reporting that the presence of atrial scar was associated with arrhythmia recurrence in patients who underwent catheter ablation (89). Moreover, since LA fibrosis is already present in the early stages of AF, late gadolinium enhancement (LGE) imaging has been proposed to discriminate patients at risk for AF (90).

Magnetic Resonance Imaging Feature Tracking (MRI-FT) is a technique which is similar to the echocardiographic speckle tracking. Regional longitudinal strain (LS) and radial motion fraction indices are measured along the atrial wall providing a quantification of standard phasic strain (91, 92). The normality range for MRI-FT-derived LA strain values have been reported on a cohort of 112 healthy volunteers (26). Data were analyzed through a commercial software (Circle Cardiovascular Imaging^®^, Calgary, Canada), and optimal intra-observer and interobserver reproducibility for all strain values was described. LA contractile strain increased significantly with age (*p* < 0.001 for all) and the LA conduit function gradually decreased (*p* = 0.02), while LA reservoir function does not vary significantly with age (*p* = 0.19). Additionally, no differences between gender were reported. Further investigations on larger cohorts of patients with different vendor softwares are still required to obtain the normal MRI-FT derived LA strain values.

### Recommendations of Guidelines

Current European and American guidelines about patients at risk of HF, subjects with cardiac structural abnormalities, or with overt HF are still based on the evaluation of standard LA parameters, mainly focused on the size of the chamber (93–103). Nevertheless, several consensuses pointed out the clinical and prognostic relevance of LA mechanics assessment (2, 85, 104–108). They also specified the need of additional standardization of parameters analysis and interpretation, and of wider prospective studies to define a specific role in clinical diagnostic work up.

The assessment of LA myocardial deformation has been recognized as a promising tool to evaluate LV diastolic dysfunction, especially in those patients with inconclusive classification based on current algorithm (107). Moreover, the consensus statement on HFpEF diagnostic workup indicates the LA mechanics as new promising markers requiring additional investigation (85). The additional value of LA function has been acknowledged in hypertrophic cardiomyopathy and HF regardless the LV systolic function, especially to investigate the burden of LA pressure overload (104–106). Moreover, the central role of LA has been fully defined in a consensus on atrial cardiomyopathies (i.e., any complex of structural, architectural, contractile, or electrophysiological changes affecting the atria with the potential to produce clinically-relevant manifestations) stressing the usefulness of myocardial deformation to assess atrial physiology and arrhythmic burden (2). Recently, a large multicenter study and an expert consensus of the EACVI on multimodality imaging in HFpEF highlighted the clinical significance of LA reservoir strain in detecting elevated LV filling pressures (108, 109). Of note, the expert consensus of the EACVI highlighted that the main usefulness of LA reservoir strain in the diagnosis of HFpEF or in the evaluation of LV filling pressures is in the setting of indeterminate echocardiographic findings (108). Moreover, the expert consensus of the EACVI remarked that the usefulness of LA reservoir strain is limited in the diagnosis of HFpEF or in the evaluation of LV filling pressures in patients with AF or with history of recent AF (108).

## LA Mechanics in HF Stages

### Stage A

Stage A is defined by any condition increasing the risk for HF but without structural heart disease or symptoms (110). All cardiovascular risk factors, including chronic kidney disease (CKD), may be considered as stage A HF, requiring specific therapeutic interventions to prevent the transition to stage B and C.

Systemic hypertension and diabetes have been shown to be associated with early reduction of all LA myocardial deformation components in subjects with normal LA dimensions (111). In hypertensive patients, the impairment of reservoir, conduit, and contractile function has been related to LV global longitudinal strain (GLS) and contractile reserve explored by dobutamine stress test and confirming the interplay between LA and LV function (112). However, the relationship between LA and LV function is stronger when atrial chamber is not dilated and seems to be less relevant when LA dilatation occurs (113, 114). A direct atrial damage, such as in diabetic myopathy, may be responsible of further chamber enlargement and function impairment independently from the degree of diastolic dysfunction (115).

Several studies showed the prognostic significance of LA mechanics in stage A HF (37, 40, 45, 116). Reservoir function has been shown to predict a composite cardiovascular end-point in a mixed population with cardiovascular risk factors and a low percentage of previous myocardial infarct and HF (116). More recently, Morris et al. investigated the additive value of LA strain analysis compared to chamber enlargement in a large population of subjects with CV risk factors. LA strain abnormalities resulted to be more prevalent than dilatation, better correlated with LV diastolic dysfunction, and independently associated with the risk of HF hospitalization during 2-years follow-up (40). The prognostic value of reservoir function was therefore confirmed in the general population and in the elderly subjects. A sub-study of Copenhagen City Heart Study considering 385 subjects without a history of cardiovascular disease showed that LA reservoir function predicted cardiovascular morbidity and mortality at the univariable analysis. However, the prognostic value was modified by sex, resulting in an independent predictor only in the female population (37). The use of reservoir to classify diastolic dysfunction in a large cohort of elderly people allowed a significant reduction of indeterminate cases, resulting in independently associated with the incidence of HF (45). In CKD where the activation of renin-angiotensin-aldosterone pathway may lead to early cardiac fibrotic remodeling, LA reservoir and enlargement emerged as early markers of cardiac involvement (117). Moreover, the reservoir function emerged as the only independent predictor of cardiovascular death and major adverse cardiovascular events in stage 3–4 CKD with higher predictive ability compared to other clinical risk scores, LV, and LA parameters (118).

*Stage A HF is characterized by early cardiac remodeling, involving LV and LA. LA mechanics reflect the degree of functional and morphological chamber adaptation, providing prognostic information of additional value with respect to standard parameters*.

### Stages B and C

Stage B and C HF have in common the element of structural heart disease while they differ for the presence of prior or current symptoms. Valvular disease is typically considered as an example of stage B due to the intrinsic high risk of HF associated with an un-prompt management. We therefore reviewed the most common diseases affecting mitral and aortic valve, along with the cardiomyopathies predisposing to stage C HF.

Moreover, we reviewed stage B and C together, as dyspnea is commonly reported in clinical practice, especially in the elderly, and is a symptom without high specificity. We also specified in Tables 2, 3 which HF stage has been considered in the reported studies.

#### Diseases Affecting Mitral Valve

Mitral valve (MV) disease corresponds to the stage B of the American Heart Association HF classification (119), according to the presence of structural heart disease potentially responsible for symptoms onset. MV disease directly expose pulmonary circulation to volumetric and/or pressure overload due to the absence of protective valves between LA and pulmonary veins. Indeed, the LA exerts a watershed effect between the MV (or the LV) and pulmonary circulation. Chronic pressure and volume overload may lead to important LA remodeling characterized by wall fibrosis, dilatation, loss of compliance, and dysfunction directly affecting pulmonary hemodynamics (120).

##### Mitral Regurgitation

The fibrotic process affecting LA secondary to MV diseases has been extensively reported in animal models. Increased levels of atrial collagen I in miniature pigs with chronic MR, mediated by the suppression of the histone deacetylase SIRT1 (silent information regulator 1), have been reported (121). Using a similar animal model, the upregulation of fibrosis-related gene transcription has also been demonstrated in LA walls, along with the increasing of angiotensin II tissue concentrations (122). In humans, analogous findings were described by Butts et al. who observed an important chymase activation in the LA walls of patients with MR, responsible for higher degrees of fibrosis, chamber enlargement, and decreased total emptying fraction (123).

Although a direct measure of LA fibrosis is very challenging in clinical practice, the early effects of such process may arise as a loss of compliance and a stiffness increase detectable through myocardial deformation analysis even before the chamber enlargement occurs. Cameli et al. demonstrated the usefulness of reservoir function, assessed through two-dimension speckle tracking echocardiography (2D-STE), to study the extent of LA fibrosis and the loss of function in 46 patients with severe MR. They showed a close negative correlation between the degree of fibrosis and the reservoir function providing histologic assessment in atrial samples obtained during cardiac surgery (124). The histologic analysis has also been used to demonstrate the correlation between the loss of reservoir function and the severity of fibrofatty myocardial replacement in 13 patients with organic MR studied with feature tracking CMR. Interestingly, the volumetric remodeling did not correlate with the degree of histological derangement that resulted with better reflected by reservoir function (125).

The remodeling process is progressive and characterized by two main phases: an early adaptation where the chamber is able to enlarge maintaining a normal SV, and a second phase where maladaptive remodeling prevails. Animal studies showed the association between MR progression and the bi-phasic atrial SV adaptation. At earlier stage, the LA enlargement favors the atrial shortening, which is essential to maintain adequate SV. Later, the SV starts to decline as the regurgitation progresses due to the shift of volume-force relationship toward a more unfavorable position (126). In humans, a significant negative correlation between ERO, reservoir, and contractile function has been reported in 102 patients with MR, including 14 patients with primary and 88 patients with secondary MR. Most of the examined cohort (84%) had a non-severe regurgitation with ERO lower to 0.2 cm^2^, demonstrating that even a mild degree of MR may lead to significant LA remodeling (127). A similar result has been recently confirmed in 80 patients with mild (*n* = 15), moderate (*n* = 20), and severe (*n* = 45) degenerative MR studied with 3D transthoracic echocardiography and vector velocity imaging. LA contractility (responsible of the active SV component) increased in response to greater LA Volume before atrial contraction (LAVpreA) up to a point beyond which the active component decreased (128). This mechanism has been further confirmed in analyzing global and regional LA mechanics in 27 patients with chronic primary MR. Compared to controls, the LA ejection force (21.5 vs. 12.3 kilodynes), the reservoir strain (32.91 ± 14.26 vs 23.14 ± 7.96%,), reservoir strain rate (2.65 ± 0.87 vs 1.62 ± 0.53 s^−1^), conduit strain rate (−2.02 ± 0.58 vs. −1.29 ± 0.59 s^−1^), contractile strain rate (−2.55 ± 1.31 vs. −1.98 ± 0.65 s^−1^), and the LA contractile tissue velocity (A′) (−5.39 ± 1.95 vs. −6.91 ± 1.80 cm/s) resulted to be all impaired, despite a similar global LA ejection fraction (31.34 vs. 29.23%), confirming the importance of active LA contraction in providing adequate LV filling (129).

**Figure 3:**
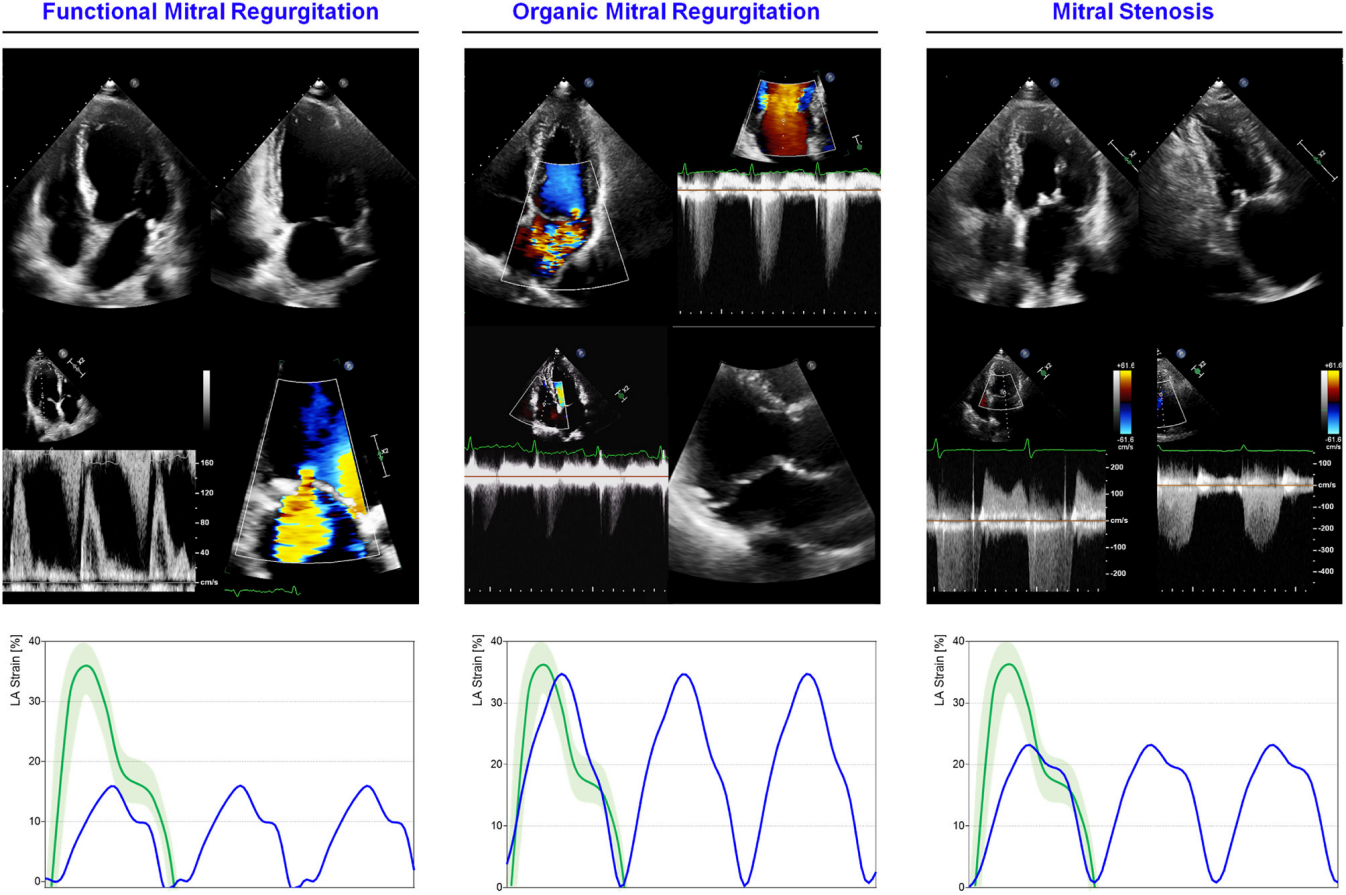
LA mechanics in mitral valve diseases: Examples of echocardiographic images and LA strain are shown for **(left)** functional MR in HF subjects with severely impaired LA function; **(middle)** severe MR secondary to bi-leaflet valve prolapse with preserved LA function; **(right)** moderate mitral stenosis with moderately impaired LA function. Strain traces are cartoons realized with real values of the reported cases. Reference values for LA strain are shown in green. Note that in organic MR, LA is still able to maintain adequate reservoir function differently from functional MR where LA function is exhausted for to the coexistence of HF. Abbreviations as in the text.

In more advanced stages of MR, all the components of LA mechanic may be impaired (Figure 3). In 43 patients with chronic primary MR, due to myxomatous valve disease, LA reservoir and contractile function, and the LA EF were impaired, whereas the conduit function was preserved. Interestingly, regional differences in LA contractility emerged in the anterior wall, probably due to the eccentricity of the systolic, anteriorly directed regurgitation jet, hitting the anterior wall and altering local wall mechanics (130). LA subclinical dysfunction has been reported in 50 patients with MV prolapse determining mild (*n* = 14), moderate (*n* = 19), and severe (*n* = 17) MR. Reservoir function resulted to be progressively impaired through the MR degrees, showing negative correlations with EROA, vena contracta, LA area, and LA volume and positive correlations with LV LS and untwisting rate (131).

The LA mechanics analysis can be of additional value for symptoms prediction in subjects with MR. Using 3D speckle tracking (3DST), Saraiva et al. assessed the correlation between LA function and pulmonary pressures in 71 patients with organic chronic MR. They reported an association between pulmonary systolic pressures, LA reservoir, and contractile function and reservoir strain rate (132). Moreover, reservoir function was correlated with a worse functional capacity and HF symptoms (NYHA III) in patients with chronic severe primary MR. Interestingly, this parameter was also linked to age and diabetes mellitus, suggesting a more accelerated LA remodeling in diabetic patients (133).

The assessment of LA mechanics has a role in prognostic stratification, identifying subjects with more advanced disease stage. In 67 asymptomatic patients with chronic primary MR, a reduced LA reservoir (<31.7%) and LV untwisting rate (< −87.9°/s) were able to identify subjects who experienced hospitalization for HF, MV surgery, or death during follow-up (24.8 ± 17 months), confirming that impaired LA mechanics and not the regurgitation severity are linked to the outcome (31). Similarly, Yang et al. examined the prognostic significance of LA mechanics in 104 patients with asymptomatic chronic severe primary MR. At follow-up (13.2 ± 9.5 months), low reservoir function (odds ratio, 3.606; 95% CI, 1.294–10.052; *p* = 0.014) and low reservoir strain rate (odds ratio, 2.857; 95% CI, 1.078–7.572; *p* = 0.035) predicted the incidence of cardiovascular mortality or MV surgery due to new-onset HF (32). Another large study on 395 asymptomatic patients with primary degenerative MR of moderate severity is in agreement. Impaired reservoir strain, LA emptying fraction, larger LA indexed volume, and lower LV strain emerged as independent predictors of cardiovascular events (AF, stroke/ transient ischemic attack, acute HF, and cardiovascular death). A global reservoir function lower than 35% emerged as the best predictor of adverse outcome during a follow-up of 3.5 ± 1.6 years (AUC of global reservoir function:0.87) (43). Similar findings have been reported in 117 subjects with moderate to severe MR due to prolapse. LA emptying fraction (HR, 2.59), reservoir strain (HR, 3.06), and contractile strain (HR, 2.01) were independently associated with cardiac surgery or all-cause mortality (38).

The serial assessment of LA mechanics may provide early insights on the chamber remodeling progression. Fifty-five patients with severe chronic MR caused by mitral prolapse or flail underwent multiple echocardiographic evaluation during a follow up of ≤ 3 months. The variation of strain rate of reservoir function from baseline to follow-up emerged as the only predictor of accelerated LA remodeling (ΔLAVi ≥ median value). Additionally, a poor baseline reservoir strain rate was significantly associated with hastened deterioration of the same parameter during the follow-up period (134).

The surgical timing for MR is another clinical context where the assessment of LA mechanics may improve patient management. In a large cohort of patients with MV prolapse and different MR degrees, total LA emptying fraction [odds ratio (OR):0.78; *p* < 0.001], reservoir function (OR:0.91; *p* = 0.028), and contractile function (OR:0.86; *p* = 0.021) emerged as independent predictors for surgery indication. Total LA emptying fraction <50% demonstrated a 91% sensitivity and 92% specificity for predicting surgical indication (135). LA reservoir strain can be used to predict outcome after surgical correction in patients with chronic severe organic MR, with values lower than 24% identifying the worse survival during a median follow up of 6.4 years (29). Similar results have been confirmed in a cohort of 71 patients with primary severe MR undergoing surgical treatment, where LA reservoir function resulted as independent predictor of clinical and functional outcome and of LA fibrosis. Considering a composite event of HF and/or cardiovascular death, 5-year event-free survival was 90 ± 5% for LA reservoir strain ≥21% and 30 ± 9% for reservoir strain <21% (*p* < 0.0001). Moreover, it was associated with an improvement of NYHA class and Borg scale after surgery (50). Preoperative reservoir strain, LAVi, and age may also predict valve repair or replacement or atrial inverse remodeling, defined as a percentage of decrease in LA volume index (136).

Differently from organic or primary MR, that is a clinical condition leading to HF if untreated, functional, or secondary MR frequently comes as a direct consequence of LV dilatation and dysfunction, further impacting on prognosis and clinical status (137–140). Rest assessment of LA mechanics in HF with severe MR has a role in prognostic stratification (Figure 3). Palmiero et al. investigated LA function in 97 patients with HFrEF and severe functional MR identifying the LA emptying fraction as an independent predictor of cardiovascular death (141).

The evaluation of LA mechanics during exercise may provide additive information, with specific insights on valvular and functional reserve (142, 143). In asymptomatic patients with degenerative MR, the presence of exercise-induced pulmonary hypertension (PH) was associated with lower reservoir function with the 26.9% threshold being independent predictor of a worse symptom-free survival (36). The difference in atrial function between primary and secondary MR has been studied by Sugimoto et al. with exercise stress echocardiography and CPET in 196 patients with primary and secondary MR, including 66 HFrEF, 19 HFpEF, and 30 HF with mid-range ejection fraction (HFmrEF). Exercise reservoir strain and contractile function were impaired in any MR type but with secondary MR exhibiting a worse atrial reservoir function. This was associated with a worsen exercise performance, limited CO increase, impaired right ventricular–to–pulmonary circulation coupling, and the highest event rate. Furthermore, LA strain during exercise was predictive of all-cause mortality and hospitalization for HF (144).

*Organic MR is characterized by progressive LA remodeling consisting of atrial wall fibrotic replacement leading to a loss of reservoir and contractile function, with prognostic significance for surgery prediction. Functional MR may present with an impairment of greater severity. The exercise-related LA functional reserve is associated with the exercise capacity and clinical outcome*.

##### Mitral Stenosis

Only fewer studies have been conducted to assess the impact of mitral stenosis (MS) on LA mechanics. Myocardial structural remodeling in MS is a known morphologic substrate of LA dysfunction that may lead to AF and adverse outcome (145). Thus, assessment of LA function in combination with LA volumetry may help guiding clinical decisions in patients with MS. Caso et al. assessed the prognostic role of LA function in 53 asymptomatic patients with rheumatic MS, finding that the best predictor of adverse events (defined as symptoms, hospitalization for cardiac cause, AF, thrombo-embolic events, valvular surgery, or percutaneous commissurotomy) at 3-year follow-up was the average LA peak systolic strain rate (cut-off value of 1.69 s^−1^), having a sensitivity of 88%, and a specificity of 80.6% (AUC:0.852) (146). Atrial mechanics analysis may be useful to predict the risk of AF in MS (Figure 3). In 81 patients with MS, an impairment of reservoir strain was observed in patient who developed arrhythmia at 5-year follow-up (13.4 ± 4.6 vs. 19 ± 5.2, *p* < 0.001) (147). Similarly, in a large cohort of asymptomatic patients with rheumatic MS that was followed up during 4 years, reservoir function was the best predictor of AF at multivariable analysis (AUC of.761 for a cut-off value of 17.4%) (30).

In order to assess possible correlation between LA function and exercise capacity in subjects with MS, Jung et al. evaluated the LA compliance (defined as 1,270 × mitral valve area by planimetry/E-wave downslope) during exercise in 33 asymptomatic patients with significant MS. Decreased LA compliance at an early stage of exercise (50 W during bicycle exercise) was an independent predictor of exercise intolerance. Moreover, a positive relationship was also noted between the chamber compliance and the pressure response of pulmonary circulation, with a more impaired LA compliance in patients who developed dyspnea at an early stage of exercise (148). Mahfouz et al. performed stress echocardiography to assess exercise intolerance in 75 patients with MV area of 1.81 ± 0.13 cm^2^ and compared them with 40 healthy control subjects. Interestingly, 44% of asymptomatic patients with mild MS had exercise intolerance, and reservoir strain was significantly associated with exercise capacity in patients with mild MS (cut-off value: reservoir strain ≤ 26.5%) (46). Similarly, Chien et al. investigated the relationship between LA deformation as measured by 2DSTE derived LA strain and HF symptoms in 69 subjects with rheumatic MS, and found that NYHA class independently correlated with LA reservoir strain and reservoir strain rate (149).

*LA reservoir function and compliance are related to exercise tolerance in MS. Reservoir function may predict the occurrence of AF*.

#### Diseases Affecting Aortic Valve

##### Aortic Stenosis

In aortic stenosis (AS), the outflow obstruction caused by a valve narrowing determines LV concentric hypertrophy and a strong predisposition to HF. The increased afterload is responsible for LV pressure overload, hypertrophy, myocardial fibrosis, impaired relaxation, and finally LA abnormal mechanics. At early stages, LA function is preserved, thus helping in the maintenance of optimal CO, but at later stages, atrial dilatation and dysfunction occur with different mechanisms (150). It has been shown that only the reservoir function impairs progressively with AS severity (151). On the contrary, contractile function seems initially enhanced in subjects with severe valvular disease without pulmonary hypertension. The enhanced contractile function acts as a compensatory mechanism driven by Frank-Starling law (LA myocytes length is augmented since LA volume is increased). Once this mechanism is exhausted, the LA contractile function starts to decline, and the chamber starts to dilate.

**Figure 4:**
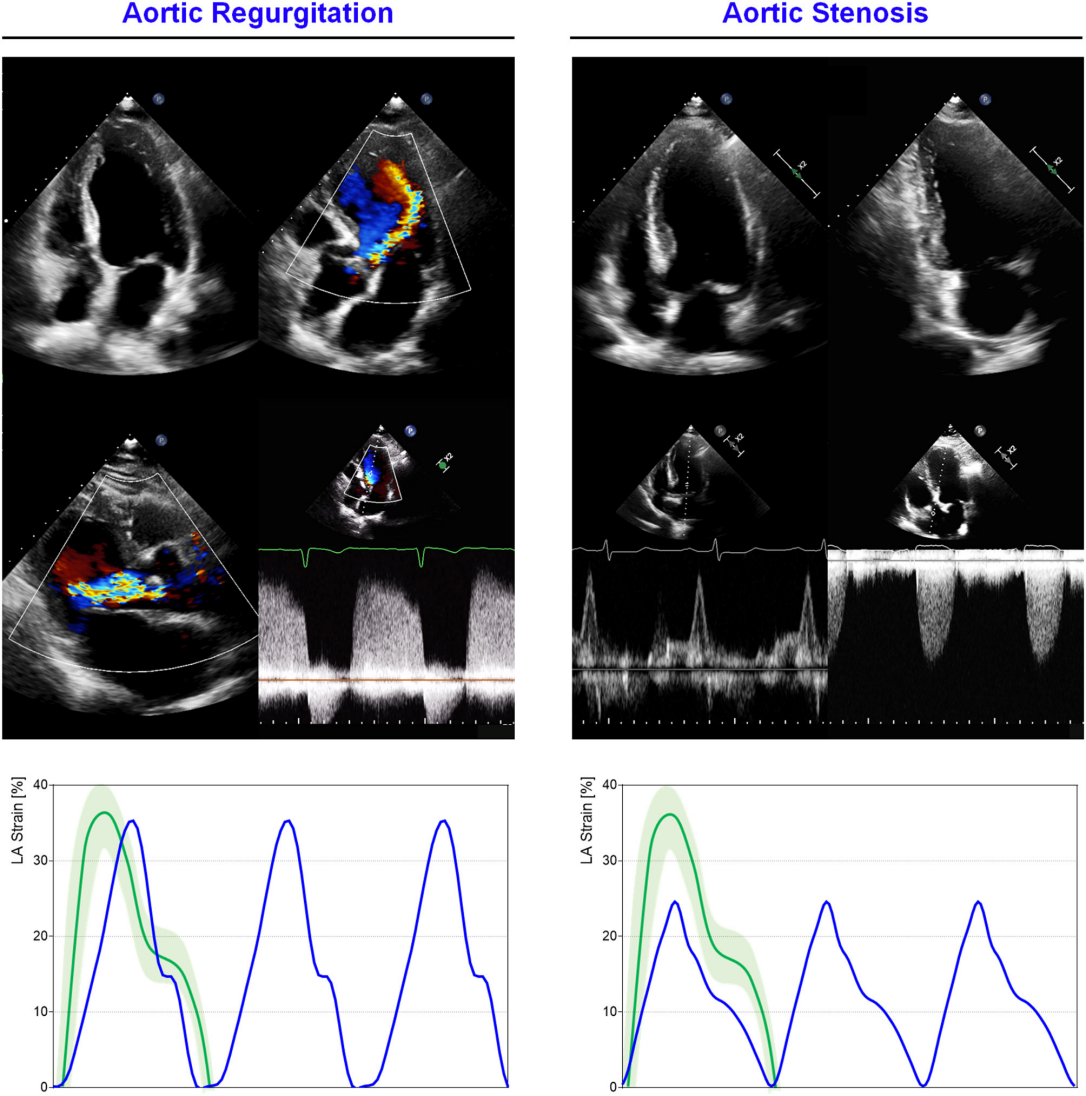
LA mechanics in aortic valve diseases: Examples of echocardiographic images and LA strain are shown for **(left)** severe aortic regurgitation (AR); **(right)** severe aortic stenosis (AS). Strain traces are cartoons realized with real values of the reported cases. Reference values for LA strain are shown in green. Note that reservoir function in AS is impaired, consistently with increased LA pressures secondary to concentric LV hypertrophic remodeling, while in AR the degree of reservoir impairment is lower. Abbreviations as in the text.

The occurrence of HF symptoms, such as dyspnea in AS, may represent the decline of clinical conditions leading to unfavorable outcome. However, in clinical practice it may be challenging to correlate the symptom with the disease progression, since the dyspnea is a common condition in elderly people, but is not necessarily of pathologic significance. LA mechanics can provide useful insights in discriminating the origin of symptoms (Figure 4). A retrospective study on 40 patients with severe AS identified contractile function, assessed through 2DSTE, as the only independent predictor of HF symptoms (dyspnea, angina, dizziness, and syncope upon exertion) at multivariate logistic regression (OR = 0.242, *p* = 0.002) including AS severity, BNP, and LV diastolic function (cut-off: LA contractile strain rate <1.01 s^−1^) (33). Recently, in a large cohort of 248 patients (202 symptomatic and 46 asymptomatic) with severe AS and preserved LV EF, the reservoir function emerged as the only parameter independently correlated with the presence of HF symptom, while LA dimensions and the echocardiographic parameters of both LV systolic and diastolic functions did not (44). Moreover, LA longitudinal strain parameters were inversely correlated with the worsening of NYHA class. These findings are consistent with the greater ability of LA mechanics in predicting prognosis when compared with LV mechanics analysis (34, 152, 153). Galli et al. (35) demonstrated that LA reservoir function (<21%) is predictive of major adverse cardiac events and HF in 128 symptomatic and asymptomatic patients with severe AS, while LV function parameters (ejection fraction and global longitudinal strain) are not. This result has been further confirmed in a recent study on 182 symptomatic and asymptomatic patients with moderate and severe AS (154). Moderate AS showed greater values of LA reservoir (23.1 vs. 13.8%, *p* < 0.001), conduit (11.5 vs. 6.5%, *p* < 0.001), and contractile function (11.5 vs. 7.1%, *p* < 0.001) when compared to severe valvular disease. On the other hand, no differences emerged when comparing LV EF, systolic, and diastolic diameters in the two populations (*p* > 0.1). The stronger prognostic and clinical significance of LA vs LV mechanics is attributable to the specific disease pathophysiology. The LV remodeling impacts on LA that dilates, enhancing reservoir and contractile function. This compensatory mechanism does not last for long time producing further pressure overload in pulmonary circulation and symptoms appearance. The inverse correlation between LA reservoir strain and PH in patients with severe AS and preserved LVEF, reflects this mechanism (155).

The importance of LA mechanics in predicting outcome in patients affected by severe AS has been corroborated by several studies reporting LA “reverse remodeling” in patients who underwent transcatheter aortic valve replacement (TAVR). LA enlargement has been reported as a marker of HF readmission after TAVR. In a large retrospective cohort of 546 patients, LA dilatation, identified by parasternal diameter (48.4 ± 7.9 vs. 43.1 ± 7.2 mm, *p* < 0.0001), was independently associated with readmission for congestive HF at 1-year follow-up (156). TAVR is generally associated with an improvement of LA mechanics, in particular, reservoir function, as assessed with 2DSTE 6 months after treatment (157). A similar improvement of LA reservoir and conduit function has been described after a longer follow up of 12 months, regardless of the AS severity, in 54 mixed severe AS (24 normal LVEF and normal flow, 16 with paradoxical low flow low gradient, and 14 with a reduced LVEF) (158).

According with the pathophysiology of AS disease, the exercise is the natural condition able to trigger LV afterload increase, LV, and LA pressure overload and therefore dynamic pulmonary congestion. While in healthy subjects, the LA reservoir and conduit functions are enhanced under stress conditions (16), the hypertrophic LV is expected to prevent relaxation and diastolic suction due to the lower compliance that chronically overloads the atrial chamber (15). Along with disease progression, LA exhausts its functional reserve leading to pulmonary congestion and symptoms appearance. Based on this pathophysiology, the rationale for dynamic assessment of LA mechanics is strong but still underused. Exercise testing with simultaneous invasive hemodynamic monitoring and Doppler echocardiography have been used to evaluate 39 patients with asymptomatic severe AS. LA size (LAVi ≥ 35 ml/m^2^) reflected the hemodynamic burden (augmented PCWP and PAP, decreased CI) and was associated at univariate Cox analysis with poor outcome (composite end-point of hospital admissions for AF, HF, and acute coronary syndrome, aortic valve replacement, and death), being a potential marker of increased hemodynamic burden during exercise. Moreover, this study suggested that when LA dilatation is overt and E/e′ is also increased, the pulmonary circulation overload is augmented, especially under stress conditions (159).

*The study of LA mechanics has been shown to be more informative compared to the LV study, especially for the prediction of HF and functional capacity. LA reservoir function is a marker of inverse remodeling occurring after AVR. The assessment during exercise is a promising frontier for the identification of asymptomatic patients at higher risk*.

##### Aortic Regurgitation

The backward flow caused by aortic regurgitation (AR) may affect LV mechanics imposing chronic volume overload, increased LV stiffness, chamber dilatation, and dysfunction. The process can persist for a long period before symptoms onset due to the ability of the LV to compensate hemodynamic overload (67). LA involvement may result from several mechanisms, including impaired LV diastolic function, fibrosis, reduced compliance, and secondary MR (160), reflecting the AR stage.

A direct correlation between LA reservoir function and PH has been reported in patients with moderate or severe AR, with a 6% increase of PH risk for each unit of LA strain decrease (151). Recently, a large study on 554 patients with bicuspid aortic valve and moderate or severe AR showed that the LA enlargement (LAVI ≥ 35 ml/m^2^) was independently associated with adverse outcome (aortic valve surgery or mortality), when compared with patients with normal LAVi (43 and 60% vs. 23 and 36%, at 1 and 5 years of follow-up, respectively, *p* < 0.001) (161). In addition to LA enlargement, its contractile function is impaired in severe AR, as reported in 65 patients scheduled for AVR and assessed with 2DSTE. The evaluation 1 year after surgery showed a reduction in LAVi (38 vs. 32 ml/m^2^, *p* < 0.001), and an improvement in both LA reservoir and contractile function (26 vs. 29% and 11 vs. 15%, respectively, *p* < 0.01) (162). These findings suggest that the volume overload imposed by AR affects LV morphology and function, along with LA mechanics, through diastolic impairment.

Although AR and AS are two distinct models of LV overload (AS determines a pressure overload while AR volume overload). They share some common effects on the LA mechanics (Figure 4). Cioffi et al. (163) compared 141 AS patients with 42 AR looking at LV geometry, LA size and function. In addition to LA size and ejection force significantly greater in AS group (Maximal LA Volume: 26 ± 7 vs 218 ± ml/m^2^, *p* = 0.0009), LA enlargement has a positive correlation with LV mass depending on LV pattern. In particular, the concentric LV pattern is related to a greater LA volume and contractile function, irrespectively of valve disease, whereas the eccentric LV geometry does not determine a relation between LA size and LV mass.

Exercise testing can unmask patients reporting to be asymptomatic. Assessment of contractile reserve is a key element to reveal subclinical LV dysfunction. The absence of contractile reserve is more predictive of the development of systolic dysfunction both at follow-up (medical therapy) and postoperatively than parameters obtained at rest in subjects with severe AR (164). Patients with severe AR may show an exercise-induced fall in LVEF due to the hemodynamic consequences of volume overload and increased afterload (165). However, the reliability of this finding in predicting outcome is controversial and it is not specifically addressed in the most recent ACC/AHA and European society of cardiology (ESC) guidelines (93, 166). If the use of exercise is logical to trigger effort-related symptoms, then, in AR, the diastole shortening induced by chronotropic response can potentially reduce the regurgitation severity, hindering AR quantification (11). AR has been evaluated through a standard cardiac 1.5-T CMR scanner under steady-state submaximal exercise and at rest. The AR % decreased during exercise from 35 (9–26, 39, 56–92) % at rest to 16 (7–25, 56–72)% during exercise, *p* = 0.003. In addition, AR at rest correlates with an increase of cardiac index during submaximal exercise (R2 = 0.64; *p* = 0.001) (167). These findings support the use of submaximal exercise to evaluate LA adaptation during effort, favoring the use of exercise echocardiography to detect an abnormal response at early stages.

*LA enlargement, loss of reservoir, and contractile function have been related to AR severity and adverse outcome in preliminary results. AVR has been shown to have a positive impact on reservoir and contractile function*.

#### Diseases Affecting Cardiac Muscle

##### Hypertrophic Cardiomyopathy

**Figure 5:**
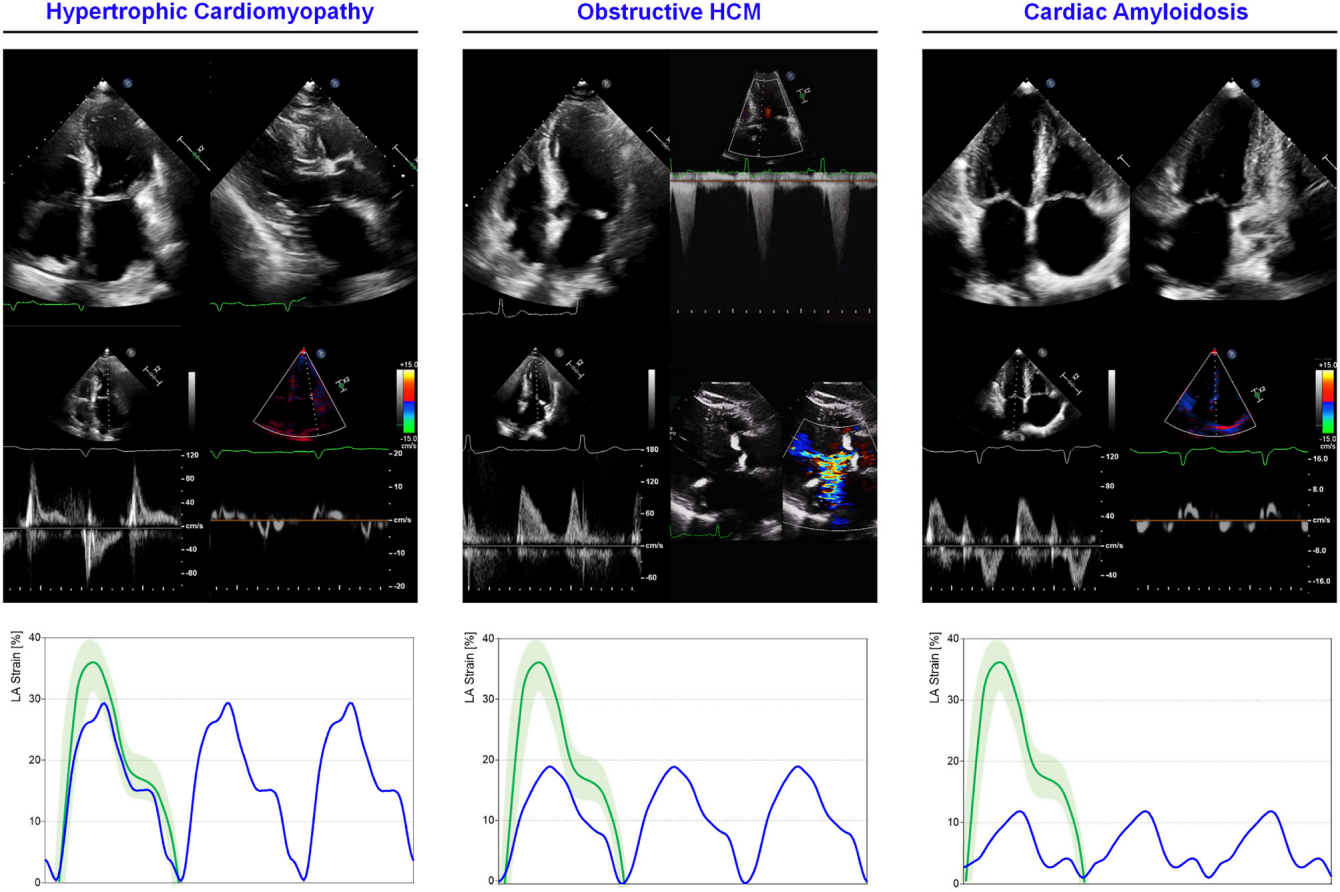
LA mechanics in cardiomyopathies: Examples of echocardiographic images and LA strain are shown for **(left)** non-obstructive HCM, **(middle)** obstructive HCM and **(right)** AL cardiac amyloidosis. Strain traces are cartoons realized with real values of the reported cases. Reference values for LA strain are shown in green. Note that non-obstructive HCM presents severe diastolic dysfunction while obstructive HCM has a greater impairment of reservoir function; AL cardiac amyloidosis presents very depressed reservoir function consistent with severe diastolic dysfunction and LA amyloid infiltration. Abbreviations as in the text.

Hypertrophic cardiomyopathy (HCM) is the most common genetic heart disorder with a prevalence of 1/200 people (168). Regardless the specific etiology, the advanced disease is characterized by LV hypertrophy, diastolic dysfunction, and increased LV filling pressures. LA adapts enlarging, increasing its contractile function until functional reserve exhausts resulting in overt dysfunction. 2DSTE has been used to describe global and phase-specific function in HCM patients (Figure 5). Total atrial deformation (defined as the sum of maximum positive and maximum negative strain during a cardiac cycle) has been shown to be significantly lower in HCM patients when compared to control subjects (169). All the three components of LA mechanics seems to be impaired in HCM patients compared to controls, in particular, strain rate at reservoir, conduit, and contractile phase has been showed to be 13, 17, and 10%, respectively lower (2.0 ± 0.6 vs. 2.3 ± 0.5 s^−1^, 1.9 ± 0.8 vs. 2.3 ± 0.7 s^−1^, 2.6 ± 0.8 vs. 3.0 ± 0.8 s^−1^; *p* < 0.05) (170). The degree of LV hypertrophy and fibrosis, assessed through CMR, is directly proportional to the degree of LA impairment. Compared with healthy controls, LA conduit function in HCM is impaired, even without extensive LGE, thus with mild or absent LV fibrosis. Conversely, LA contractile function is reduced only in HCM patients with a higher degree of fibrosis, leading to a more advanced diastolic dysfunction, consequent to LA enlargement and functional impairment (171). A more advanced atrial myopathy and disfunction play a specific role in determining symptoms. Contractile function, explored with 2DSTE, emerged as the only independent predictor of HF symptoms with a cut-off of −0.92 s^−1^ for contractile strain rate (sensitivity: 75%, specificity: 83%, area under the curve:0.83) in a series of 37 HCM patients, with enlarged atria compared to controls (28). In less advanced disease, LA dysfunction can be already present even if the chamber size is still in the normal range. In non-obstructive HCM patients with normal LA size and contractile function, reservoir and conduit components resulted impaired when compared with healthy controls. This finding is consistent with a lower degree of LV fibrosis, earlier disease stage and more preserved atrial physiology (172). On the other hand, LA function further lowers if LV outflow tract obstruction is present, as demonstrated comparing 50 obstructive, 50 non-obstructive and 50 healthy patients studied with feature-tracking CMR. The presence of obstruction has a great impact on LA EF (42.3 ± 8 vs. 47.2 ± 9%; *p* = 0.004), reservoir strain (14.5 ± 4 vs. 17.7 ± 5%; *p* = 0.002), strain rate (0.59 ± 0.2 vs.73 ± 0.2 s^−1^; *p* = 0.001), contractile strain (6.1 ± 2 vs 7.5 ± 3%; *p* = 0.01), and strain rate (−0.44 ± 0.1 vs −0.58 ± 0.25 s^−1^; *p* = 0.004) when compared to the non-obstructive HCM group (173) (Figure 5).

Different studies evaluated the prognostic role of LA function assessment in HCM patients. An early report identified total LA strain (the sum of reservoir and contractile function) as the strongest predictor of short term (12-month) outcome of death or hospitalization for cardiovascular causes, with the optimal cut-off of 21% (odds ratio 0.858, 95% CI 0.771–0.954, *p* = 0.005). The same parameter was also informative for the occurrence of AF requiring hospitalization (odds ratio 0.853, 95% CI 0.748–0.972, *p* = 0.017) (27). The long-term prognostic significance of LA function was further demonstrated in a large series of HCM subjects and controls, followed-up for 55 months. Total LA strain (−17.4%; *p* < 0.001), LV outflow tract obstruction (*p* < 0.001), and E/e' (10.3; *p* = 0.02) emerged as independent predictors of the composite endpoint of all-cause death, heart transplantation, LV assist device implantation, and clinical worsening (41). Recently, the prognostic value of LA function assessment has been addressed with the use of CMR. During a long follow up-up of 40.9 months, 59 patients with HCM (19.7%) out of 359 experienced the composite endpoint of cardiovascular death, resuscitated cardiac arrest, sudden cardiac death aborted by appropriate ICD discharge, and HF hospital admission. Reservoir and conduit components (HR, 0.94 and 0.89; *p* = 0.019 and 0.006, respectively) emerged as independent predictors of outcome, also after correcting for the extent of LV fibrosis, confirming the prognostic significance of LA mechanics and expanding the clinical applications of CMR-based myocardial deformation analysis (47).

Throughout the clinical history of HCM, the occurrence of AF marks a critical point potentially impacting on HF symptoms and prognosis. The study of LA mechanics can be informative on the occurrence of new-onset arrhythmia. In large series of HCM patients studied with 2DSTE, LA volume and reservoir strain were able to predict the occurrence of new-onset AF during a follow-up of 4.8 ± 3.7 years. The presence of LA reservoir strain >23.4% predicted a superior 5-year AF-free survival (98 vs. 74%, *p* = 0.002) (174). This finding has been recently confirmed by Vasquez et al. who reported a similar threshold for reservoir function (> 23.8%) and a threshold of 10.2% for conduit function to predict event-free survival during a follow-up of 5.83 ± 0.3 years (events defined as heart failure, stroke, and death) (42).

In HCM patients, LA mechanics may partially restore after surgical and non-surgical therapy, as observed in a subset of 20 patients with obstructive HCM who underwent septal myomectomy, after which LAEF (41.6 ± 13 vs 48.4 ± 10%; *p* =0.006), reservoir (14.1 ± 6 vs. 17.3 ± 7%; *p* = 0.01) and contractile function (6.8 ± 4 vs 9.8 ± 5%; 0.0001) increased at CMR (173). The reversibility of reservoir impairment after treatment provides an additional explanation of the clinical response to the septal reduction. These findings, obtained with non-invasive approach, are consistent with previous data reporting an improvement of LV relaxation with consequent increase of LV passive filling volume, decrease in LA volumes, ejection force (defined as: 0.5 x 1.06 x mitral annulus area x (peak A^2^), in kdyne), kinetic energy (0.5 x 1.06 x LA SV x (peak A^2^) in kerg), and a parallel reduction of NYHA class, with longer exercise duration (*p* < 0.05) (175).

*Left Atrium (LA) mechanics progressively disrupt in HCM, reflecting the disease severity and fibrosis extension. Reservoir function is the first to decline, especially when HCM determines LVOT obstruction. All the components of atrial function are able to predict the outcome, representing valid prognostic markers*.

##### Restrictive Cardiomyopathy

Despite restrictive cardiomyopathies (RCMs) are known to be the least common among the heart muscle diseases, they include a wide group of conditions characterized by different pathogenesis, clinical presentation, diagnostic workflow, treatment, and prognosis (176). Some possible etiologies include infiltrative disorders, such as amyloidosis or sarcoidosis, storage disorders like Fabry disease, and idiopathic RCM. The ventricular myocardium generally presents with increased stiffness, responsible for the characteristic diastolic dysfunction, elevation of filling pressures, and atria dilation. Systolic function is usually preserved until the late stages of the disease. During exercise, the poor compliance of the ventricles hinders the rapid venous return, resulting in an important rise of the filling pressures and in a limited increase of SV (177). The progressive atrial enlargement may contribute to the onset of HF symptoms, atrial arrhythmias, or secondary atrioventricular valvular regurgitation.

In cardiac amyloidosis (CA), the LA dysfunction has been repeatedly studied. Loss of all components of atrial function characterizes cardiac amyloidosis regardless of the etiology (light chain, mutant or wild-type transthyretin). However, among the amyloidosis subtypes, transthyretin amyloidosis (ATTR) wild type generally present the worst reservoir and contractile function. Of note, even after adjusting for LA size, LV EF, and LV filling pressures, all LA function components are generally impaired in CA patients, when assessed with 2DSTE (Figure 5) (39, 178, 179). A significative alteration in the reservoir and contractile function has also been recorded with real-time 3D echocardiography, and there is evidence that, according to the progression of the disease, the LA mechanics gradually undergoes greater impairment (39, 179). Recently, myocardial deformation of both LA and LV have been shown to be linked to prognosis in CA. In particular, a reservoir function <13.2% demonstrated a 7.5-fold increased risk of all-cause mortality over a median follow-up of 5 years (95% CI 3.8–14.7, *p* < 0.001) in a cohort of 136 patients (48). Remarkably, in a large population of >900 subjects with ATTR, the LA stiffness, estimated as the ratio between E/e' and reservoir function, has been shown to be an independent marker of prognosis, after adjustment for the main echocardiographic and clinical parameters. In the same population, the presence of LA electro-mechanical dissociation (absence of valid mechanical contraction despite sinus rhythm at ECG) emerged as a distinct phenotype with impaired outcome, similar to subjects with AF (49). LA mechanics are therefore significantly impaired in infiltrative disease, with a growing evidence of a direct LA involvement contributing to the severe mechanics impairment.

Unlike cardiac amyloidosis, primary LA involvement is less clear in sarcoidosis. Small studies using 2DSTE reported impairment of LA reservoir function in sarcoidotic subjects when compared to controls, with a negative correlation with the disease stage. However, it is unclear if the loss of function is a consequence of a primary atrial involvement or simply a consequence of LV dysfunction (180, 181).

Left atrium (LA) reservoir function, evaluated by 2DSTE, has been reported to be significantly impaired in patients with Fabry disease even in presence of normal echocardiographic assessment (182, 183). It is still unclear if these findings can be primarily explained by the increase of LV filling pressures or by the direct depositions of sphingolipids in the LA. However, LA stiffness seems to be an early marker of atrial remodeling, already altered before the occurrence of LV hypertrophy (184). Conversely, a relevant impairment in atrial conduit function has been reported only in presence of LVH, and this may be justified by a more advanced LV diastolic dysfunction (184). Using CMR with T1 mapping to classify patients with Fabry disease, Bernardini et al. reported a progressive impairment of reservoir function, assessed with FT-CMR, according with the reduction of T1 mapping (index of subclinical disease) or the presence of LV hypertrophy (overt heart involvement) (185). Nevertheless, in another study, significant differences in the three LA function components were found only in Fabry disease with significant LV hypertrophy (186).

*Despite the heterogeneity of restrictive diseases, the impairment of reservoir function has been reported as a common pathophysiological element. In aTTR-CA, the estimation of LA stiffness emerged as a strong and independent prognostic marker*.

##### Dilated Cardiomyopathy

Dilated cardiomyopathy (DCM) is a complex pathological condition coursing with HF and representing the most common indication for heart transplantation worldwide (187), and it is characterized by the presence of LV dilatation and systolic dysfunction, worsened by abnormal LV filling pressures and functional mitral regurgitation in the most advanced stages (188, 189). At earlier stages, LA contractile function is augmented to maintain adequate LV filling, but later it decreases as a consequence of the increased afterload.

In a series of 160 DCM and 154 ischemic patients, studied with 2DSTE and CPET, LA reservoir and contractile functions were significantly reduced in the DCM group, with LA lateral wall reservoir and LA volume predictive of peak VO_2_ (both *p* < 0.001) (190). Similar findings were reported by Cao et al. in 32 ischemic, 26 DCM, and 32 control patients where reservoir and contractile functions were more impaired in DMC subjects (191).

The prognostic role of contractile function loss and LA dilatation has been demonstrated in a large cohort of patients with DCM (192) and recently confirmed with the use of CMR (193). LA maximal volume (LA_max_) resulted to be effective in predicting the occurrence of a composite endpoint including death or heart transplantation in a population of 337 patients with DCM who were followed-up for a mean period of 41 ± 29 months. Notably, patients with an increased LA volume (LAVi> 68.5 ml/m^2^) had a risk ratio of 3.8 compared with those with a preserved LA volume (194). In line with these results, indexed LA area assessed with standard echocardiography (with an optimal cut-off > 13 cm^2^/m^2^) emerged as the strongest index associated with the same composite outcome both in a univariate and a multivariate model in 275 DCM patients, whom events were recorded over a mean follow-up of 67 months (HR 6.58, 95% CI 2.43–17.86, *p* < 0.001 and HR 3.2, 95% CI 1.06-9.23, *p* = 0.038, respectively) (195).

The reliability of these results is confirmed by similar findings obtained assessing the LA geometry with CMR. In particular, LAVi resulted to be an independent predictor of a composite endpoint including all-cause mortality or cardiac transplantation evaluated in 483 consecutive patients affected by non-ischemic DCM who were prospectively followed-up over a median period of 5.3 years (HR per 10 ml/m^2^ 1.08, 95% CI 1.01–1.15, *p* = 0.022). Furthermore, patients with an increased LAVi (> 72 ml/m^2^) showed a three-fold elevated risk of death or transplantation (HR 3.00, 95% CI 1.92–4.70, *p* < 0.001). LAVi was also independently associated with the secondary composite endpoints of cardiovascular mortality or cardiac transplantation (HR per 10 ml/m^2^ 1.11; 95% CI 1.04–1.19, *p* = 0.003), and HF death, HF hospitalization, or cardiac transplantation (HR per 10 ml/m^2^ 1.11; 95% CI 1.04–1.18; *p* = 0.001) (196).

Of note, some LA morphological and functional parameters demonstrated to have a prognostic role when evaluated under stress. In particular, in 84 DCM patients studied with dobutamine stress echocardiography and followed-up for a mean period of 17.0 ± 11.8 months, LAVi (HR 1.060, 95% CI, 1.035–1.087; *p* < 0.001) besides the variation of systolic LA strain (HR, 0.971, 95% CI, 0.946–0.996, *p* = 0.02) and the variation of passive LA strain (HR 0.942, 95% CI, 0.914–0.971, *p* < 0.001) emerged as independent predictors of cardiovascular events in two different multivariate Cox models. Interestingly, including LA strain parameters at rest and under dobutamine into multivariate Cox analysis provides an incremental benefit in predicting adverse cardiovascular events (197).

*Left atrium (LA) enlargement and loss of reservoir function occurring in DCM reflects the disease severity and have a prognostic significance for composite endpoints. The use of dobutamine to test the LA functional reserve may have an incremental value in risk stratification*.

#### Heart Failure With Reduced, Mildly Reduced, and Preserved EF

**Figure 6:**
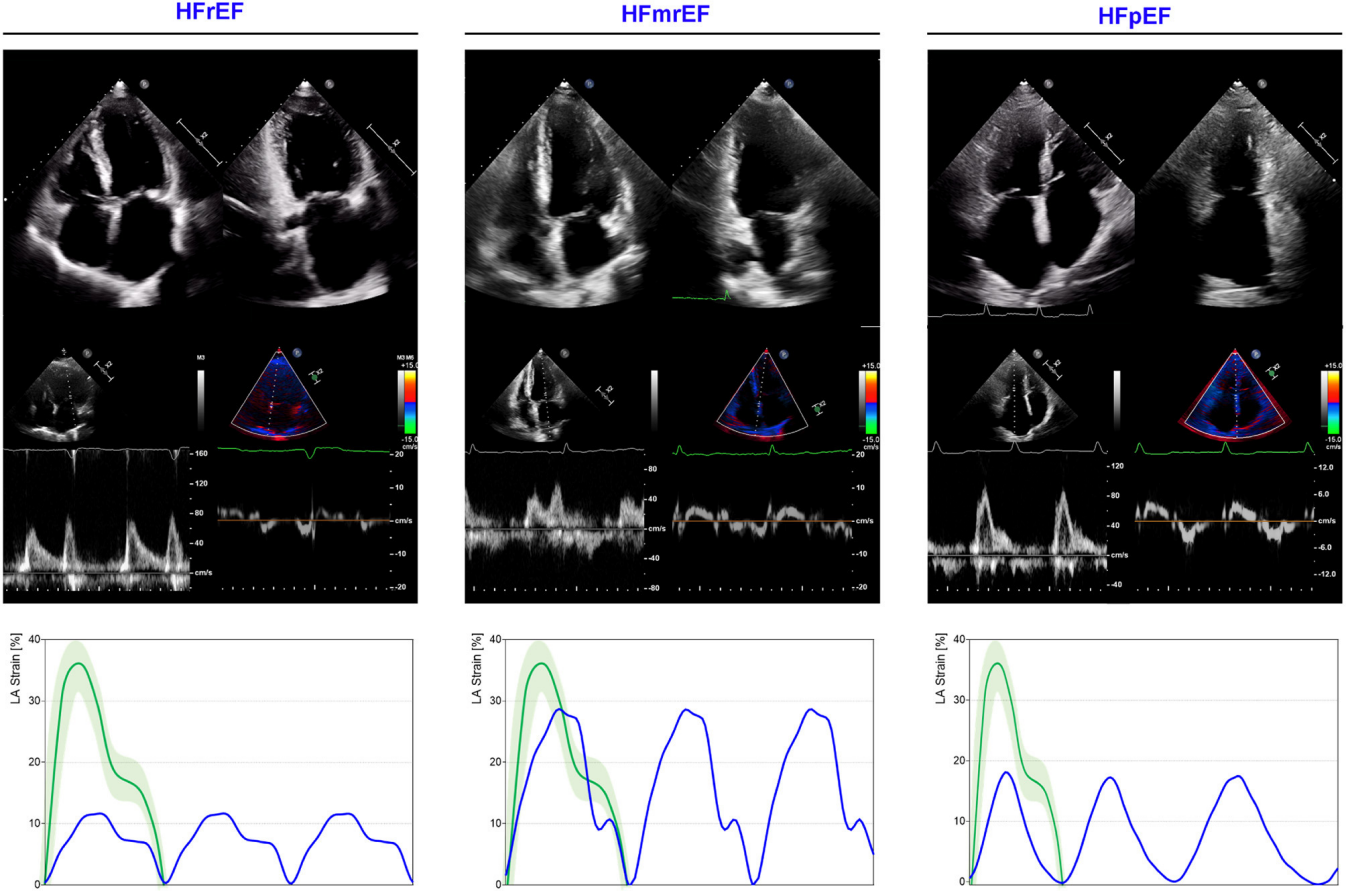
LA mechanics in HF subtypes. Examples of echocardiographic images and LA strain are shown for HFrEF **(left)**, HF with mid-range ejection refraction (HFmrEF; **middle)** and HF with preserved ejection refraction (HFpEF; **right**). Strain traces are cartoons realized with real values of the reported cases. Reference values for LA strain are shown in green. Note that HFrEF has a very depressed LA function when compared with HFmrEF (expression of greater disease severity), while HFpEF presents a significantly impaired reservoir function (without contraction component for the presence of atrial fibrillation), similar to that observed in HFrEF. Abbreviations as in the text.

Left atrium (LA) represents the physiological escape for the augmented LV filling pressures occurring in every type of HF syndrome (Figure 6). According with the HF duration and onset velocity, LA reacts to pressure overload adapting its dimensions (198), function, and compliance, and plays a dominant role in the disease progression (199). The remodeling ability of LA directly impacts on pulmonary circulation, eventually leading to pulmonary capillary involvement, pulmonary artery hypertension (200), and RV failure (201). Nevertheless, differences exist in the remodeling process occurring in HF with reduced vs. preserved LV EF. A greater chamber enlargement and a greater increase in LA stiffness and pressures have been described in HF with reduced ejection fraction (HFrEF) and HF with preserved ejection fraction (HFpEF), respectively through the use of right heart catheterization (RHC) and echocardiography in a large cohort of mixed HF patients (202). In HFrEF, LA dilatation is directly related to LV disease progression, primarily through the hemodynamic effects, while in HFpEF different disease pathways (i.e., inflammation and direct atrial myopathy) may interact resulting in a more complex LA remodeling (203). Nevertheless, the LA dysfunction similarly impacts on pulmonary circulation in both phenotypes, resulting strictly correlated with pulmonary vascular disease and RV dysfunction (202, 204, 205). The loss of atrial compliance, reflected by an increased stiffness (non-invasively estimated as the ratio between E/e' and reservoir function), has been showed to be predictive of HF hospitalization and cardiac death in HFrEF and HFmrEF patients (206). LA stiffness estimation has the advantage of exploring the mechanical behavior of the chamber, combining the expansibility properties with the degree of LV pressure overload.

The non-invasive estimation of LA pressure is a cornerstone of echocardiographic evaluation, providing information on the hemodynamic conditions of LA-pulmonary circulation unit (107). An integrative approach, considering diastolic parameters, LA dimensions, and mechanics may improve the hemodynamic assessment and provide additional prognostic information. The assessment of LA reservoir function with 2DSTE improves the detection of LV diastolic dysfunction in subjects with preserved LV EF and LA size, improving the current diastolic function algorithms (45) and resulting associated with a higher risk of HF hospitalization, even after adjusting for age and sex (40).

In the context of HFrEF, LA reservoir function is an independent predictor of adverse outcomes (a combined end-points of all-cause mortality and HF hospitalization) in stable patients, with an incremental predictive value compared to standard parameters of LV function (51, 207) and LA dilatation (208). The loss of reservoir function has a prognostic significance also in the context of acute HF in subjects with preserved sinus rhythm. Moreover, reservoir function correlates with functional impairment and presents a better ability in predicting poor quality of life when compared to LA volume and LV dysfunction (67). The tight relationship between LV systolic and LA reservoir function (being the LV base downward displacement one of the main determinants) has been raised as a matter of concern about the independent prognostic significance of atrial deformation (209). However, its incremental predictive value, compared to standard parameters of LV function (51, 207) and LA dilatation (208), has been demonstrated. As for systolic and diastolic components of LV mechanics, LA function reflects several factors and interactions. Indeed, the parameters describing the single phases should not be considered *per se* but rather as a part of a more complex system.

The interest on LA mechanics has grown especially for the investigation of physiology, for early diagnosis and prognostic stratification of HFpEF, based on the key role of the atrium. Longitudinal data from a large study cohort showed that the loss of atrial reservoir function is associated with increased risk of HF hospitalization, even after adjusting for clinical risk factors, NTproBNP and echocardiographic parameters, in patients with coronary artery disease and preserved LV EF (65). Similarly, in patients with definite diagnosis of HFpEF enrolled in the TOPCAT trial, LA reservoir function emerged as a valid predictor of HF hospitalization (210). The effort tolerance, the most common symptom in HFpEF, is strictly modulated by reservoir function, being associated with abnormal pulmonary vascular resistance and impaired functional capacity (peakVO_2_) (211). The LA emptying function and LV filling properties are both correlated with NTproBNP levels in HFpEF subjects, as shown in a RELAX trial sub-study (212).

In a large cohort of 363 symptomatic patients, LA reservoir (cut off: <24.5%) function and compliance (estimated as the ratio between reservoir and E/e', cut off: 3) outperformed E/e', LA enlargement, tricuspid regurgitation velocity, LV hypertrophy and LV global longitudinal strain (GLS) in diagnosing HFpEF, using exercise RHC as a diagnostic standard (53). The impairment of reservoir function has been linked with the progression of AF burden in HFpEF. Combining RHC and echocardiographic evaluation in a cohort of 285 HFpEF patients, the presence of a reservoir function <31.5% and a compliance <5.7%/mmHg has been associated, respectively, with a hazard ratio (HR) of 6.8 and 6.0 for the progression toward worse AF stage (55). This finding supports a model of electro-mechanical coupling, expression of a remodeling process where reservoir and contractile function are influenced also by electrical properties.

Along with the remodeling process affecting LA in HFpEF, the occurrence of mitral regurgitation (MR) represents a further step associated with a greater hemodynamic severity and a poorer functional capacity. Interestingly, the presence of LA disfunction (defined as LA reservoir <24.5%) remains an independent predictor of HF or cardiovascular death, even after adjusting for age, gender, BMI, LV EF, and the presence of MR itself, confirming the prognostic importance of the chamber.

The assessment of rest LA reservoir function has been validated in the diagnostic workup of HFpEF. Ye et al. tested the predictive role of rest reservoir function in identifying abnormal exercise-induced LV filling pressure (as defined by 2016 AHA guidelines for diastolic evaluation) in a cohort of 669 subjects. The addition of LA reservoir function to the currently recommended diagnostic work-up improved the diagnostic accuracy (AUC from 0.71 to 0.80, *p* = 0.01) with a reported 28% higher odds of developing elevated exercise LV filling pressure per 1% of reservoir function decrease (213).

The interaction between LA function and exercise capacity represents another area of interest in all HF phenotypes. During physical effort, LA plays a major role in ensuring adequate and rapid LV filling. The abnormal rise of LV end-diastolic pressure during exercise, typical of both HFrEF and HFpEF, prevents the physiological emptying of LA, leading to a rise in atrial pressures during diastole. The interplay between atrial function and effort tolerance has been variably shown in all HF phenotypes, using different approaches, including standard echocardiography, myocardial deformation and radionuclide assessment (19, 214, 215). A large study on 486 subjects, symptomatic for chest pain or dyspnea, with preserved LV ejection fraction, explored the determinants of exercise capacity with echocardiography. LA reservoir function, E/e', age, male gender and BMI emerged as independent predictors of effort tolerance (19). A similar result has been recently reported in HFpEF patients of a German registry, where a LA reservoir <22% was able to predict impaired functional capacity after adjustment for common variables and log-NTproBNP (216). The reservoir function is not exclusively linked to functional capacity. Von Roeder et al. investigated the role of the different components of LA mechanics reporting a strong association between impaired conduit function and reduced early LV filling in HFpEF, by using a multimodality approach. The loss of conduit function limits the early LV filling and therefore the SV, one of the CO component, resulting in restricted peak VO_2_ during exercise (171).

The study of LA mechanics at rest may predict the exercise response in HF patients, as demonstrated in a cohort of 164 HF patients (56% with preserved EF) who underwent to rest and exercise RHC. LA reservoir function (with a threshold of 21 and 17% in HFrEF and HFpEF, respectively) predicted rest or exercise elevated pulmonary capillary wedge pressure (PCWP) with higher accuracy than recommended algorithm (AUC: 0.80 vs.69, *p* < 0.001) (52). The strong correlation between LA function and the degree of pulmonary congestion has been recently confirmed by Telles et al. (54) using simultaneous RHC and strain analysis in 49 HFpEF and 22 subjects with non-cardiac dyspnea. Reservoir and contractile LA function correlated with exercise PCWP, remaining independent predictors after adjustment for other variables, and showing a good diagnostic accuracy with a reservoir cut-off of 33%. LA reservoir function directly reflects the pulmonary hemodynamic status and the response to the unloading effect of diuretic therapy. Deferm et al. (7) showed a strong and rapid improvement of reservoir function during and after acute pulmonary decongestion in 31 acute HFrEF patients with invasive pressure monitoring and serial echocardiographic assessment. Remarkably, the contractile function slowly recovered during the observation period, suggesting the persistence of a great stunning condition predominantly affecting atrial contraction.

**Figure 7:**
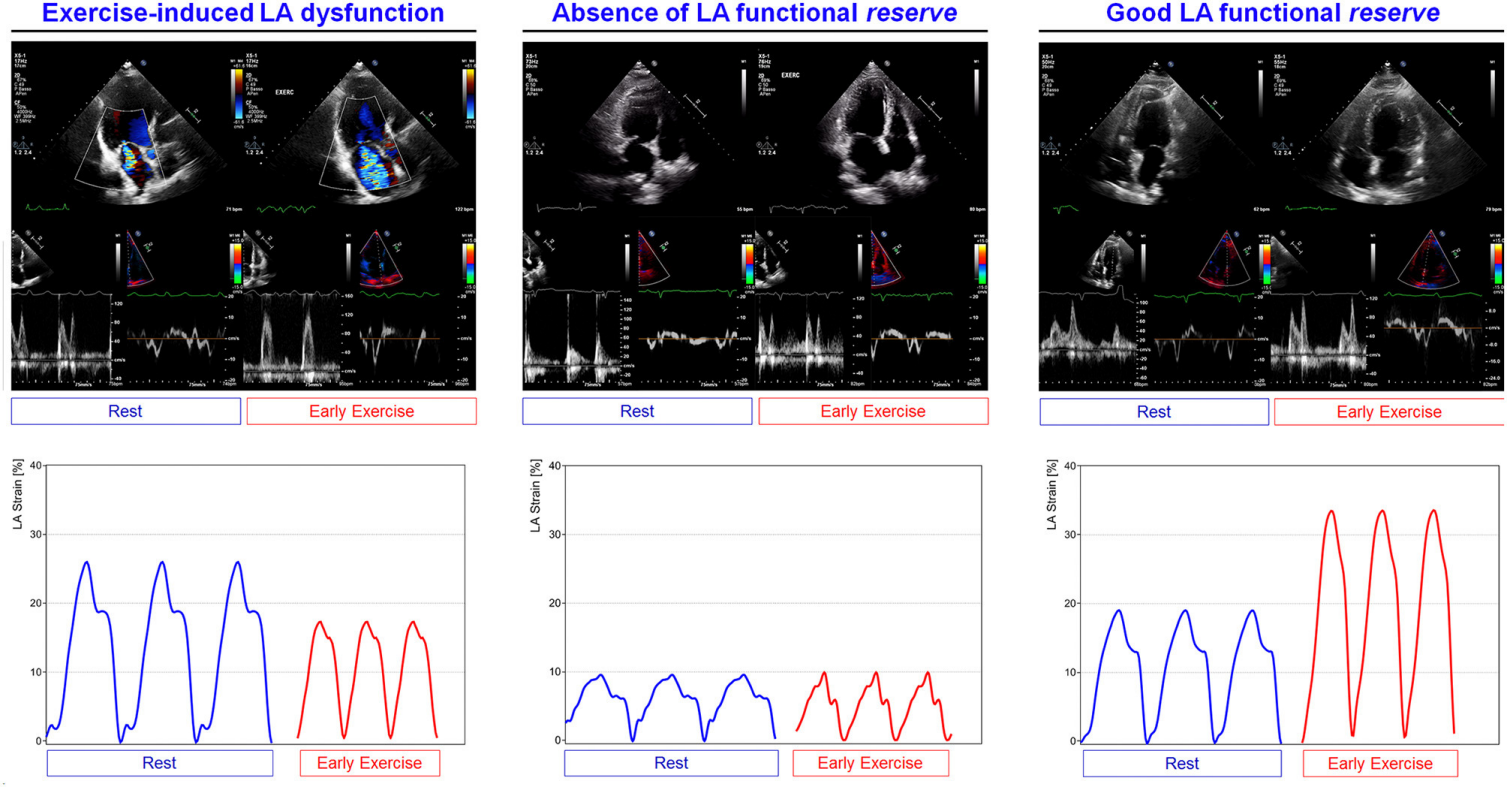
LA functional reserve assessed during exercise in HFrEF. Examples of rest-stress traces of patients with different severity of HFrEF. **(left)** Exercise-induced LA dysfunction; **(middle)** absence of LA functional reserve in severely impaired LA mechanics; **(right)** good LA functional reserve. Note that during early stage of exercise, LA mechanics can improve or reduce according with the presence of functional reserve, expression of multiple factors influencing LA function. In the first case on the left, the presence of exercise-induced mitral regurgitation produces additional volume overload responsible of further reservoir function worsening. Abbreviations as in the text.

The use of stress test to explore the chamber reserve represents the emerging frontier of LA mechanics assessment. This approach can acquire a clinical relevance in specific context, such as the HFpEF diagnostic workup, where the diagnostic gold standard (invasive hemodynamic at rest and during exercise) requires an uncommon level of expertise, still not widely available (93, 166). The use of cardiovascular imaging in dynamic conditions—to test LA reserve—may provide a more effective recognition of pathological response than a rest-limited assessment. Obokata et al. (217) explored the use of passive leg lift in testing the LA reserve to discriminate HFpEF from hypertensive patients. They confirmed that LA dilatation in HFpEF occurs to maintain an adequate SV at rest. Nevertheless, during passive volume overload, HFpEF presents with a reduced reservoir and contractile function, provoking a blunted SV increase during exercise. Remarkably, the use of such a simple stressor, better discriminated HFpEF from hypertensive patients, showing additional diagnostic value compared to conventional parameters. The significance of LA mechanics during exercise has been reported in a large population of mixed HF patients who underwent exercise-echocardiography and cardiopulmonary exercise test (CPET). The study of atrial myocardial deformation during the early phase of exercise showed that peak SV, CO, and cardiac power output were all associated with a greater reservoir function reserve, triggered by exercise. As suggested by the data collected during rest evaluation, a deficient reservoir reserve during exercise affects the LV filling and the backward flow to pulmonary circulation leading to blunted CO and pulmonary circulation retrograde overload (218). Interestingly, the loss of LA reservoir reserve has been observed irrespectively from LV EF and other hemodynamic factors, being a marker for the occurrence of HF hospitalization and death. Finally, the presence of functional MR in HFrEF subjects was associated with a further reduction in exercise-related LA reservoir function, confirming the additional detrimental effect of volume overload leading to earlier dilatation and exhaustion of atrial function (144), as represented in Figure 7.

The absence of LA reservoir function reserve, in both HFrEF and HFpEF, has been studied with respect to functional phenotypes and right ventricle-pulmonary circulation unit, using a combined exercise-echocardiography and CPET approach. Compared to control subjects, a limited or absent reservoir function reserve was observed in HFpEF and HFrEF, respectively, during early exercise phase. Remarkably, the exercise-induced LA reservoir function correlated with TAPSE/PAPS ratio, a marker of right ventricle-pulmonary circulation coupling, and with VE/VCO_2_, an index of ventilatory efficiency, in both types of HF syndrome (219).

*Left atrium (LA) adaptation to abnormal pressure overload imposed by HF is a crucial determinant of hemodynamic and functional conditions. Reservoir function is a key parameter to address the global function of the chamber, the presence of functional reserve and to use in diagnostic workup and prognostic stratification*.

## Conclusions

Left atrium (LA) remodeling plays a central role in cardiac diseases due to the ability of the chamber in adapting to abnormal hemodynamic conditions, generated by the underlying disease, and to protect pulmonary circulation. The assessment of LA mechanics (especially reservoir function), with 2DSTE or FT-CMR, is very informative on the stage of disease progression and on the risk stratification. The evaluation under stress conditions, mainly during physical exercise, is a great potential for the additional insights on the LA functional reserve.

## Author Contributions

FB, AM, MF, GG, and NV organized database and wrote the manuscript. All authors contributed to manuscript revision, read, and approved the submitted version.

## Funding

The article has been funded by Gruppo Ospedaliero San Donato Foundation.

## Conflict of Interest

The authors declare that the research was conducted in the absence of any commercial or financial relationships that could be construed as a potential conflict of interest.

## Publisher's Note

All claims expressed in this article are solely those of the authors and do not necessarily represent those of their affiliated organizations, or those of the publisher, the editors and the reviewers. Any product that may be evaluated in this article, or claim that may be made by its manufacturer, is not guaranteed or endorsed by the publisher.
