# Supplementary material for: Cardiac Imaging for the Assessment of Left Atrial Mechanics Across Heart Failure Stages
Source: Front Cardiovasc Med. 2022 Jan 13;8:750139. doi: 10.3389/fcvm.2021.750139 (PMC8792604; doi:10.3389/fcvm.2021.750139)
Supplement: Supplementary file 1 [file Table_1.docx]

**Supplementary Table 1**. Overview of additional parameters describing LA mechanics with reference values according to age/gender (when available) and related to the imaging technique.

| Parameter | Definition | Technique | Normal Range | Ref |
| --- | --- | --- | --- | --- |
| Left Atrial Expansion Index (LAEI) | Relative LA volume increase during the reservoir phase | 2D - 3D TTE | 190 (165-342)%, 211 (183-252)%, 212 (165-259)%, 191 (166-244)%, 173 (151-208)%, 157 (146, 268)% for groups 18-29, 30-39, 40-49, 50-59, 60-69 and over 70 years [median (25th percentile - 75th percentile)] | (1) |
| Passive Emptying Fraction (PEF) | (Maximal LA Volume - LA volume at the onset of P wave on ECG) / Maximal LA Volume | 2D TTE | 43.0±10.3 % [mean ± SD] | (2) |
|  |  | CMR | 44 (33-56) %, 41 (29-53) %, 37 (26-49) %, 34 (22-46) %, 30 (19-42) %, 27 (15-39) % for groups aged 20-29, 30-39, 40-49, 50-59, 60-69 and 70-79 years, respectively [mean (95 % confidence interval)] | (3) |
| Active Emptying Fraction (AEF) | (LA volume at the onset of P wave on ECG - Minimal LA Volume) / LA volume at the onset of P wave on ECG | 2D TTE | 43.1±9.4 % [mean ± SD] | (2) |
|  |  | CMR | 32 (19-46) %, 34 (20-47) %, 35 (22-49) %, 37 (23-50) %, 38 (25-51) %, 39 (26-53) % for groups aged 20-29, 30-39, 40-49, 50-59, 60-69 and 70-79 years, respectively [mean (95 % confidence interval)] | (3) |
| Total Emptying Fraction (TEF) | (Maximal LA Volume - Minimal LA Volume) / Maximal LA Volume | 2D TTE | 71.3 (67.3–74.9) %, 66.7 (62.8–72.4) %, 64.0 (58.1–69.5) % for groups aged 20-40, 40-60 and over 60 years, respectively [median (25th percentile - 75th percentile)] | (2) |
|  |  | 3D TTE | 58.4 (53.1–63.1) %, 57.1 (52.2–61.3) %, 55.6 (50.6–60.4) % for groups aged 20-40, 40-60 and over 60 years, respectively [median (25th percentile - 75th percentile)] | (2) |
|  |  | CMR | 62 (51-74) %, 61 (50-72) %, 60 (48-71) %, 58 (47-70) %, 57 (45-68) %, 55 (44-67) % for groups aged 20-29, 30-39, 40-49, 50-59, 60-69 and 70-79 years, respectively [mean (95% confidence interval)] | (3) |
| Reservoir Strain Function | Myocardial deformation measured as difference of strain value at mitral valve opening minus ventricular end- diastole | 2D STE | 46.8 (42.3–52.4) %, 40.9 (35.4–46.1) %, 35.5 (30.9–41.9) % for groups aged 20-40, 40-60 and over 60 years, respectively [median (25th percentile - 75th percentile)] | (2) |
|  |  | 3D STE | 29.2±8.3% [mean ± SD] | (4) |
|  |  | MRI-FT | 38.48 ± 9.31 % [mean ± SD] | (5) |
| Contractile Strain Function | Myocardial deformation measured as difference of strain value at ventricular end-diastole minus the onset of atrial contraction | 2D STE | 15.6 (11.9–19.0) %, 16.3 (13.2–19.6) %, 16.8 (13.6–21.4) % for groups aged 20-40, 40-60 and over 60 years, respectively [median (25th percentile - 75th percentile)] | (2) |
|  |  | 3D STE | 9.9 ± 5.2 % [mean ± SD] | (4) |
|  |  | MRI-FT | 11.72 ± 2.97 %, 12.56 ± 3.54 %, 13.62 ± 3.14 %, 15.05 ± 4.87 %, 18.02 ± 3.28 % for groups aged 18-29, 30-39, 40-49, 50-59, 60-79 years, respectively [median (25th percentile - 75th percentile)] | (5) |
| Conduit Strain Function | Myocardial deformation measured as difference between the strain value at the onset of atrial contraction and the atrial valve opening | 2D STE | 30.6 (26.8–36.5) %, 24.1 (19.7–29.3) %, 18.6 (14.7– 22.6) % for groups aged 20-40, 40-60 and over 60 years, respectively [median (25th percentile - 75th percentile)] | (2) |
|  |  | MRI-FT | 25.66 ± 7.89 %, 26.63 ± 8.19 %, 28.52 ± 9.03 %, 21.02 ± 6.58 %, 22.51 ± 7.99 % for groups aged 18-29, 30-39, 40-49, 50-59, 60-79 years, respectively [median (25th percentile - 75th percentile)] | (5) |
| Reservoir Strain Rate | (Positive) peak strain rate during reservoir phase | MRI-FT | 1.93 ± 0.54 s-1 [mean ± SD] | (5) |
| Contractile Strain Rate | (Negative) peak strain rate during contraction phase | 2D STE | −1.31 (−1.99 - −0.95) s-1 [median (25th percentile - 75th percentile)] | (2) |
|  |  | MRI-FT | −1.89 ± 0.52 s-1, −1.69 ± 0.54 s-1, −2.00 ± 0.43 s-1, −2.17 ± 0.56 s-1, −2.56 ± 0.72 s-1 for groups 18-29, 30-39, 40-49, 50-59, 60-79 years [mean ± SD] | (5) |
| Conduit Strain Rate | (Negative) peak strain rate during conduit phase | MRI-FT | −2.39 ± 0.65 s-1, −2.09 ± 0.67 s-1, −2.26 ± 0.67 s-1, −1.85 ± 0.60 s-1, −1.95 ± 0.80 s-1 for groups 18-29, 30-39, 40-49, 50-59, 60-79 years [mean ± SD] | (5) |

TTE: transthoracic echocardiography; CMR: cardiovascular magnetic resonance; 2D STE: 2D speckle-tracking echocardiography; 3D STE: 3D speckle-tracking echocardiography; MRI-FT: magnetic resonance imaging - feature tracking; PAWP Pulmonary Artery Wedge Pressure.

**REFERENCES – Supplemental Material**

1. Badano LP, Miglioranza MH, MihǍilǍ S, Peluso D, Xhaxho J, Marra MP, Cucchini U, Soriani N, Iliceto S, Muraru D. Left atrial volumes and function by three-dimensional echocardiography: Reference values, accuracy, reproducibility, and comparison with two-dimensional echocardiographic measurements. Circ Cardiovasc Imaging (2016) 9:1–12. doi:10.1161/CIRCIMAGING.115.004229

2. Sugimoto T, Robinet S, Dulgheru R, Bernard A, Ilardi F, Contu L, Addetia K, Caballero L, Kacharava G, Athanassopoulos GD, et al. Echocardiographic reference ranges for normal left atrial function parameters: Results from the EACVI NORRE study. Eur Heart J Cardiovasc Imaging (2018) 19:630–638. doi:10.1093/ehjci/jey018

3. Maceira AM, Cosin-Sales J, Prasad SK, Pennell DJ. Characterization of left and right atrial function in healthy volunteers by cardiovascular magnetic resonance. J Cardiovasc Magn Reson (2016) 18:1–16. doi:10.1186/s12968-016-0284-8

4. Nemes A, Kormányos Á, Domsik P, Kalapos A, Lengyel C, Forster T. Normal reference values of three-dimensional speckle-tracking echocardiography-derived left atrial strain parameters (results from the MAGYAR-Healthy Study). Int J Cardiovasc Imaging (2019) 35:991–998. doi:10.1007/s10554-019-01559-z

5. Truong VT, Palmer C, Wolking S, Sheets B, Young M, Ngo TNM, Taylor M, Nagueh SF, Zareba KM, Raman S, et al. Normal left atrial strain and strain rate using cardiac magnetic resonance feature tracking in healthy volunteers. Eur Heart J Cardiovasc Imaging (2020) 21:446–453. doi:10.1093/ehjci/jez157
